# Selective USP7 inhibition elicits cancer cell killing through a p53-dependent mechanism

**DOI:** 10.1038/s41598-020-62076-x

**Published:** 2020-03-24

**Authors:** Nathan J. Schauer, Xiaoxi Liu, Robert S. Magin, Laura M. Doherty, Wai Cheung Chan, Scott B. Ficarro, Wanyi Hu, Rebekka M. Roberts, Roxana E. Iacob, Björn Stolte, Andrew O. Giacomelli, Sumner Perera, Kyle McKay, Sarah A. Boswell, Ellen L. Weisberg, Arghya Ray, Dharminder Chauhan, Sirano Dhe-Paganon, Ken C. Anderson, James D. Griffin, Jianing Li, William C. Hahn, Peter K. Sorger, John R. Engen, Kimberly Stegmaier, Jarrod A. Marto, Sara J. Buhrlage

**Affiliations:** 1 Department of Cancer Biology and the Linde Program in Cancer Chemical Biology, Dana-Farber Cancer Institute, Boston, MA USA; 2 Department of Biological Chemistry and Molecular Pharmacology, Harvard Medical School, Boston, MA USA; 3 Department of Systems Biology and Laboratory of Systems Pharmacology, Harvard Medical School, Boston, MA USA; 4 Department of Chemistry and Chemical Biology, Northeastern University, Boston, MA USA; 5 Department of Pediatric Oncology, Dana-Farber Cancer Institute and Boston Children’s Hospital, Boston, MA USA; 6 Dr. von Hauner Children’s Hospital, Department of Pediatrics, University Hospital, LMU Munich, Munich, Germany; 7 The Broad Institute of MIT and Harvard University, Cambridge, MA USA; 8 Department of Medical Oncology, Dana-Farber Cancer Institute, Boston, MA USA; 9 Department of Medicine, Harvard Medical School, Boston, MA USA; 10 Harvard College, Cambridge, MA USA; 11 Department of Chemistry, University of Vermont, Burlington, VT USA; 12 The LeBow Institute for Myeloma Therapeutics and Jerome Lipper Myeloma Center, Dana-Farber Cancer Institute, Boston, MA USA; 13 Department of Oncologic Pathology and Blais Proteomics Center, Dana-Farber Cancer Institute, Boston, MA USA; 14 Department of Pathology, Brigham and Women’s Hospital and Harvard Medical School, Boston, MA USA

**Keywords:** Small molecules, Target validation

## Abstract

Ubiquitin specific peptidase 7 (USP7) is a deubiquitinating enzyme (DUB) that removes ubiquitin tags from specific protein substrates in order to alter their degradation rate and sub-cellular localization. USP7 has been proposed as a therapeutic target in several cancers because it has many reported substrates with a role in cancer progression, including FOXO4, MDM2, N-Myc, and PTEN. The multi-substrate nature of USP7, combined with the modest potency and selectivity of early generation USP7 inhibitors, has presented a challenge in defining predictors of response to USP7 and potential patient populations that would benefit most from USP7-targeted drugs. Here, we describe the structure-guided development of XL177A, which irreversibly inhibits USP7 with sub-nM potency and selectivity across the human proteome. Evaluation of the cellular effects of XL177A reveals that selective USP7 inhibition suppresses cancer cell growth predominantly through a p53-dependent mechanism: XL177A specifically upregulates p53 transcriptional targets transcriptome-wide, hotspot mutations in *TP53* but not any other genes predict response to XL177A across a panel of ~500 cancer cell lines, and *TP53* knockout rescues XL177A-mediated growth suppression of *TP53* wild-type (WT) cells. Together, these findings suggest *TP53* mutational status as a biomarker for response to USP7 inhibition. We find that Ewing sarcoma and malignant rhabdoid tumor (MRT), two pediatric cancers that are sensitive to other p53-dependent cytotoxic drugs, also display increased sensitivity to XL177A.

## Introduction

Ubiquitination is a post-translational modification that regulates myriad fundamental cellular processes, most notably protein degradation^1^, and is implicated in numerous disease settings, including cancer^2,3^, infection^4^, and neurodegeneration^5^. In particular, the ubiquitin-proteasome system (UPS) has become a target of interest in oncology, as proteasome inhibitors and ligands targeting the E3 ubiquitin ligases are approved as cancer therapies^6,7^. As the enzymes responsible for proteolytically cleaving ubiquitin moieties, deubiquitinating enzymes (DUBs) regulate virtually all physiological and pathophysiological processes^1,8–10^. Ubiquitin-specific peptidase 7 (USP7) is one of the most widely studied DUBs, and it has been associated with multiple substrates, cellular pathways, and disease states. USP7 was first discovered as an interacting partner and stabilizer of the Herpesvirus E3 ligase ICP0^11^. Since then, USP7 has also been reported to interact with and regulate numerous mammalian E3 ligases, epigenetic modifiers, and transcription factors, among other targets^12^.

Of these potential substrates, USP7’s stabilization of MDM2, the E3 ligase for p53, has garnered the most interest from a mechanistic and therapeutic standpoint given the role of p53 as tumor suppressor across many cancer types. Although many cancers are driven by mutant *TP53*, roughly half of all adult malignancies and 95% of pediatric cancers harbor intact WT *TP53* and may benefit from therapeutic approaches that stabilize p53^13,14^. Indeed, MDM2 inhibitors such as idasanutlin and the dual MDM2/MDM24 inhibitor ATSP-7041 are currently undergoing clinical evaluation^15,16^, supporting investigation of additional p53 stabilizing strategies such as USP7 inhibition. However, USP7’s promiscuity has raised questions over the relative importance of p53 in its overall cellular function. USP7 has more than 20 reported substrates (see recent reviews^12,17,18^), several of which (PTEN^19^, FOXO4^20^, N-Myc^21^, PCNA^22^, Claspin^23^, and others) play a key role in proliferation and tumorigenesis. Indeed, USP7 has now been proposed as a therapeutic target independent of *TP53* mutational status in multiple cancers including bortezomib-resistant multiple myeloma^24^, neuroblastoma^21^, T-cell acute lymphoblastic leukemia^25^, and acute myeloid leukemia^26^. These findings have spurred interest in the development of specific USP7 inhibitors, as there now appear to be several cancer indications that may benefit from USP7 modulation. There have been a number of small molecule USP7 inhibitors reported to date^26,27^, and these compounds have consistently demonstrated the ability to stabilize p53 protein levels *in cyto*, although they do not exhibit p53-dependent growth suppression^26,28^. These findings have largely been credited to USP7’s role in other cellular pathways, although they could also be due to unidentified off-targets of these compounds.

We recently reported XL188^29^, a potent and selective USP7 inhibitor that, in contrast to other reported small molecules, induces p53-dependent growth suppression in *TP53*-WT Ewing Sarcoma cell lines^30^. This somewhat surprising finding suggested to us that the functional significance of the USP7-p53 axis in Ewing Sarcoma, and perhaps cancer more broadly, is not completely understood and that improved research tools are needed to enable reliable on-target analysis of USP7 biology. To that end, we undertook the development of novel irreversible XL188 analogs that could be comprehensively profiled for USP7 selectivity and then used as chemical tools to elucidate cellular responses to selective USP7 inhibition.

Here, we report the discovery of a 340 pM irreversible inhibitor of USP7, XL177A. XL177A labels the catalytic cysteine, C223, of USP7 with exquisite selectivity for USP7 across the DUBome and human proteome. Evaluation of XL177A and its enantiomer, XL177B, as a negative control, across a broad panel of 484 cancer cell lines and in a series of targeted confirmatory studies, found that *TP53* mutational status predicted response to selective USP7 inhibition across multiple cancer lineages. We also found that, in *TP53*-WT cell lines, *TP53* knockout (KO) rescues XL177A and *USP7*-KO-mediated growth suppression. Overall, our findings demonstrate that covalent targeting of the active-site is a viable strategy for development of DUB inhibitors, provide a well-validated chemical tool for investigation of USP7 biology, and demonstrate that p53 is a relevant biomarker for cellular responses to USP7 inhibition.

## Results

### Rational design of irreversible USP7 inhibitor XL177A

**Figure 1:**
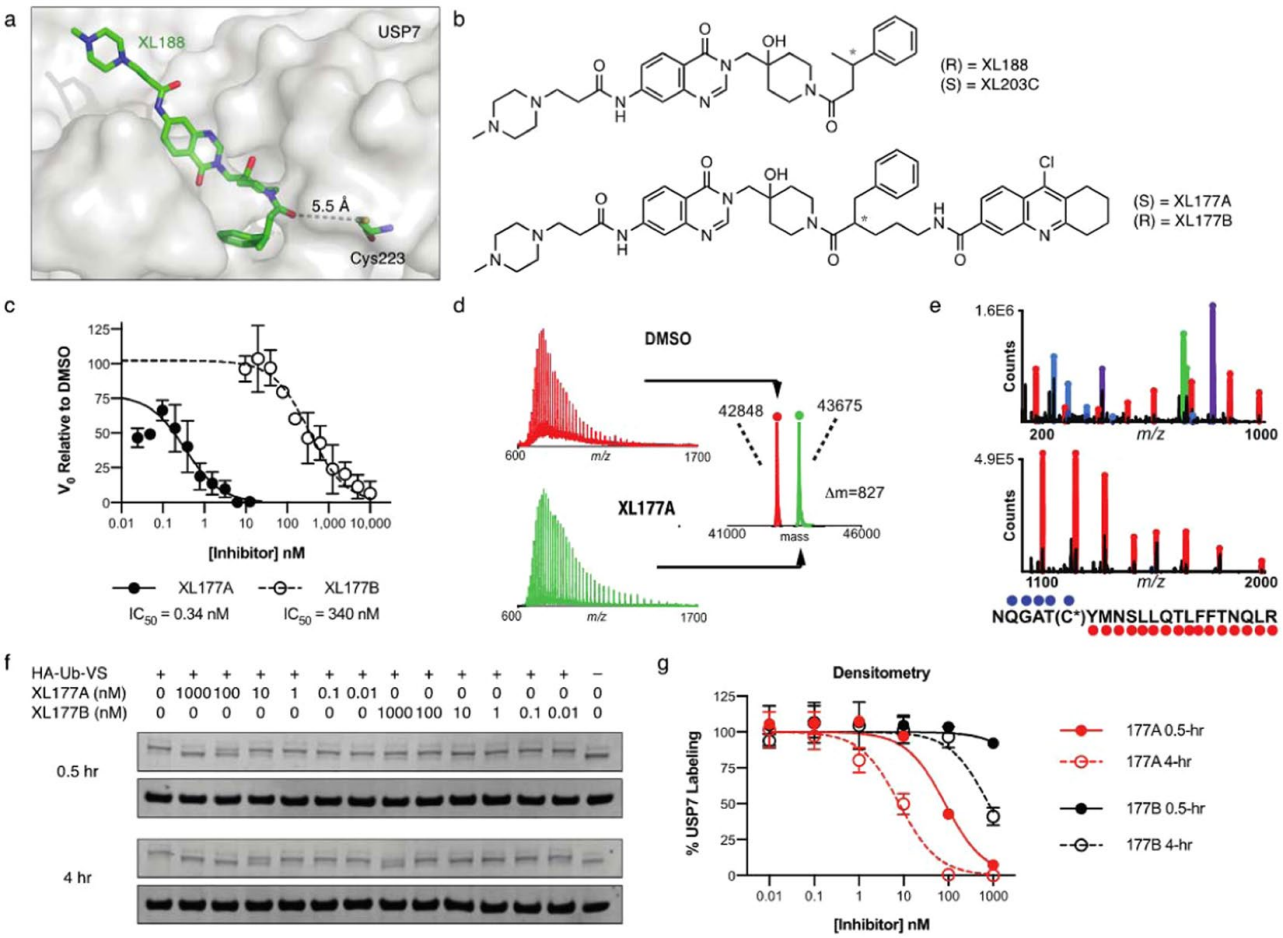
Design of a potent irreversible USP7 inhibitor. (**a**) Co-crystal structure of XL188 bound to the USP7 catalytic domain, highlighting the ligand’s solvent accessibility and distance to the catalytic cysteine (PDB: 5VS6). (**b**) Chemical structures of XL188, XL177A, and XL177B. (**c**) IC_50_ plots of full-length USP7 cleavage of Ub-AMC following 6-hour pre-treatment with XL177A or XL177B (n = 3 experimental replicates, error bars = 95% CI). (**d**) Intact MS spectra of USP7 catalytic domain treated with DMSO (red) or a 2.5-fold molar excess XL177A for 15 minutes (green), showing single labeling of USP7 with a mass shift corresponding to the inhibitor (n = 2 experimental replicates). **(e**) MS-MS spectra of USP7 catalytic domain treated with 2.5-fold molar excess XL177A for 15 minutes. The XL177A-labeled cysteine (C223) is indicated in the peptide sequence shown. (**f**) Western blots showing USP7 labeling by the DUB ABPP HA-Ub-VS in whole cell lysate after 30-minute or 4-hour pre-treatment with XL177A or XL177B. Full blots are presented in Fig. S14. (**g**) Densitometry from F (n = 2 experimental replicates, error bars = SD).

**Figure 2:**
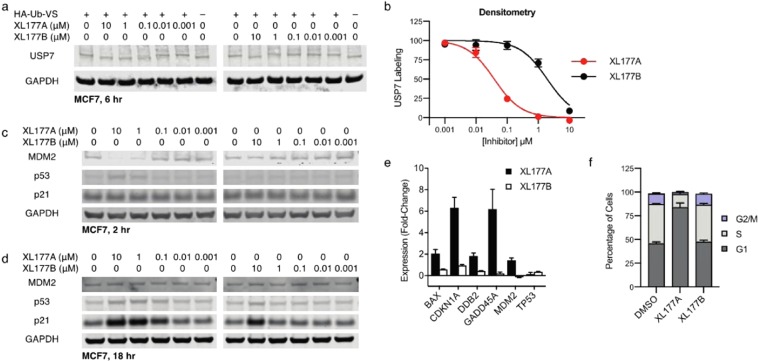
XL177A is a potent USP7 inhibitor and p53 stabilizer *in cyto*. (**a**) Western blots showing USP7 labeling by HA-Ub-VS after 6-hour cell treatment with XL177A or XL177B. (**b**) Densitometry from A (n = 2 experimental replicates, error bars = SD). (**c**) Whole cell lysate Western blots of MCF7 cells after 2-hr treatment with XL177A or XL177B (n = 2 experimental replicates). Full blots are presented in Fig. S14. (**d**) Whole cell lysate Western blots of MCF7 cells after 16-hr treatment with XL177A or XL177B (*n* = 2). (**d**) Quantitative real-time PCR of MCF7 cells treated for 24 hours with 1 μM XL177A or XL177B (n = 2 experimental replicates with 3 technical replicates each, error bars = SEM). E) Cell cycle analysis based on propidium iodide staining of MCF7 cells after 24-hr treatment with 1 μM XL177A or XL177B (n = 3 experimental replicates, error bars = SD).

We recently reported the structure-guided development of XL188, a noncovalent inhibitor of USP7 that binds in the thumb-palm cleft that guides the ubiquitin C-terminus into the active site. Specifically, a co-crystal structure of XL188 and the USP7 catalytic domain shows the compound bound within the S4-S5 pocket of enzyme about 5 Å removed from the catalytic cysteine (Fig. 1a). The proximity of the compound to the catalytic Cys residue suggested that elaboration of XL188 may yield a new series of covalent (irreversible) USP7 inhibitors. Chemical synthesis and biochemical characterization of >50 XL188 analogs (*Liu et al*., in preparation) culminated in development of XL177A as a highly potent and selective irreversible inhibitor of USP7 (Fig. 1b). In an enzymatic assay using full-length USP7 and fluorogenic substrate ubiquitin-7-amino-4-methylcoumarin (Ub-AMC), XL177A inhibited USP7 with an IC_50_ of 0.34 nM (Fig. 1c). An accurate measure of the enzyme inactivation rate (k_inact_) and inhibition constant (K_I_) for XL177A was not feasible due to rapid and complete labeling of USP7 by the compound. However, determination of these parameters for XL041, a precursor compound containing the same electrophilic warhead, confirmed an irreversible mode of inhibition for the compound series (Fig. S1). A covalent binding mode was also confirmed for XL177A using mass spectrometry. We incubated purified USP7 catalytic domain with vehicle (DMSO) or XL177A for 15 minutes and analyzed samples using capillary electrophoresis-mass spectrometry (CE-MS). Quantitative labeling of USP7 by XL177A with a mass shift corresponding to inhibitor mass minus HCl, was observed (Fig. 1d). MS/MS analysis confirmed binding to the catalytic residue, C223 (Fig. 1e). An analog of XL177A without the chloride leaving group, XL058, was unable to label the USP7 catalytic domain by MS (Fig. S2), and no mass shift was detected when XL177A was incubated with USP7 C223A (Fig. S3). To test USP7 target engagement in a cellular context, we used competitive activity-based protein profiling (ABPP) with MCF7 crude cell extracts and the DUB activity-based probe (ABP) hemagglutinin (HA)-ubiquitin vinylmethylsulfone (HA-Ub-VS): XL177A inhibited HA-Ub-VS labeling with IC_50_s of 85 and 8 nM following 30 min and 4 hr compound preincubations, respectively (Fig. 1f,g). We also employed live cell treatment and competitive ABPP to demonstrate that XL177A inhibits USP7 *in cyto*, with an IC_50_ of 39 nM after 6 hr treatment (Fig. 2a,b). The enantiomer of XL177A, XL177B (Fig. 1b), also demonstrated time-dependent USP7 inhibition, but with ~500-fold less potency against USP7 in experiments using purified enzyme, crude lysate, and live cell treatment (Figs. 1c,e, 2a,b). XL177B is thus a useful matched control for assessing the USP7-specific effects of XL177A.

To further confirm inhibition of cellular USP7, we determined the impact of XL177A/B on the HDM2-p53 signaling axis, which is a well validated target of USP7 DUB activity. As expected, treatment of MCF7 cells, which express WT *TP53*, with XL177A induced rapid degradation of HDM2 within 2 hours, followed by increases in p53 and downstream p21 protein levels (Fig. 2c,d) and stimulating transcription of p53 target genes related to both cell cycle arrest (*CDKN1A* and *GADD45A*) and apoptosis (*BAX* and *DDB2*) (Fig. 2e). Indeed, 1 μM XL177A induced complete G1 arrest in MCF7 cells after 24 hours (Figs. 2f and S4). In contrast, negative control XL177B did not exert any of the same effects except at 10 μM, where it demonstrated USP7 inhibition and on-target cell signaling effects (Figs. 2c–e, and S4). Due to negative feedback signaling, whereby p53 transcriptionally upregulates *MDM2*, after 24 hours of treatment with XL177A, p53 and p21 protein levels remained high, but MDM2 protein levels matched DMSO control (Fig. 2d).

### XL177A binds USP7 in the XL188 binding pocket

**Figure 3:**
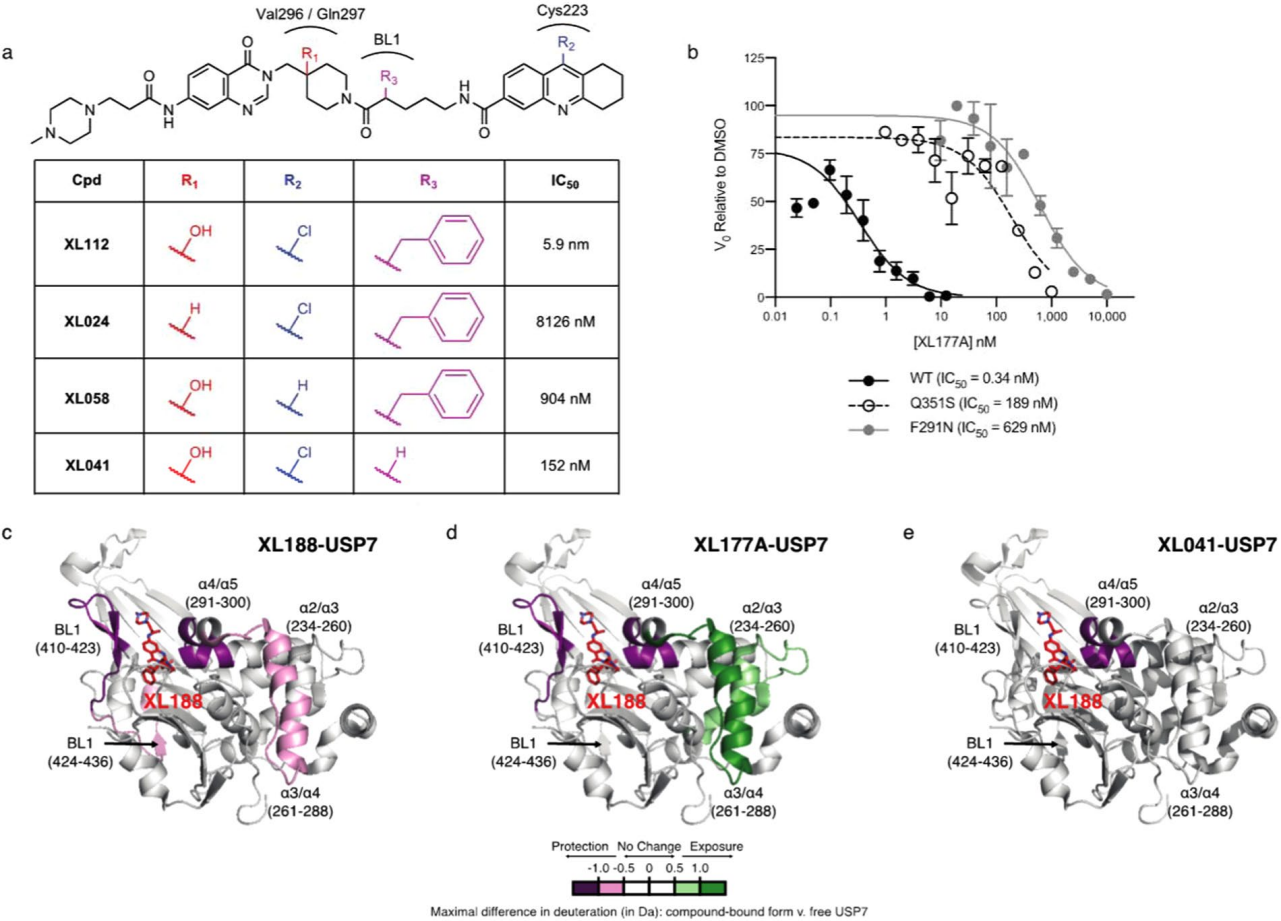
XL177A has a similar USP7 binding mode to XL188. (**a**) Structure-activity-relationship of XL177A showing the potency of the indicated compounds against full-length USP7 in Ub-AMC assays (n = 2 or 3 experimental replicates). (**b**) Comparative Ub-AMC IC_50_ curves of USP7-WT, USP7-Q351S, and USP7-F291N after 6-hr pre-incubation with XL177A (n = 2 experimental replicates, error bars = 95% CI). (**c–e**) Structure of USP7 CD highlighting regions with increased (green) or decreased (purple) hydrogen exchange after treatment with XL188 (**c**), XL041 (**d**), and XL177A (**e**) (PDB: 5VS6; n = 2 experimental replicates).

XL188 binds the S4-S5 pocket of USP7 (Fig. 1a), and we hypothesized that XL177A was still binding this pocket. Unfortunately, extensive efforts to crystallize the USP7-XL177A complex for structure determination by X-ray were unsuccessful. We thus investigated binding mode using structure-activity-relationship (SAR) studies, USP7 mutant enzyme studies, hydrogen-deuterium exchange mass spectrometry (HDX), and molecular dynamics (MD) simulations. The 4-hydroxy-piperidine group of XL188 forms hydrogen-bonding interactions with the sidechain carboxylic group of USP7 Q297 and the peptide backbone of V296 and is required for USP7 inhibition: XL024 (IC_50_ = 8 μM), which has a hydrogen atom instead of this hydroxyl group, is ~1,000-fold less potent than XL112 (a racemic mixture of XL177A and XL177B, IC_50_ = 0.0059 μM) and is ~10-fold less potent than XL058 (IC_50_ = 0.904 μM), which lacks the Cys223-reactive chloro atom (Fig. 3a and Table S1). In addition, two XL188-resistant USP7 mutants, F291N and Q351S^27,29^, are inhibited by XL177A with 100-fold loss in potency compared to wild-type enzyme (Figs. 3b and S5). To gain more direct structural information about the interaction of XL177A with USP7, we performed HDX to monitor changes in protein dynamics^31^. Both XL188 and XL177A strongly protected the BL1 and α-4/5 loops surrounding the S4-S5 pocket from exchange (Fig. 3c,d and S6). Consistent with the benzyl moiety of XL177A being buried in the S4 pocket, an XL177A analog lacking the benzyl group (XL041) does not protect β-sheet residues 410–423 that engage the benzyl group on XL188, which may explain the 100-fold loss of potency for XL041 compared to XL177A (Fig. 3a,e, and S6). While the regions of protection for XL188 and XL177A are similar we observed enhanced exchange in the region from α2 to α4 of USP7, putatively due to changes in dynamics resulting from covalent bond formation. These helices likely became destabilized upon XL177A binding, leading to exposure of the backbone hydrogens. We note that this XL177A-induced change in USP7 conformation may help explain why crystallization efforts with this compound were unsuccessful, with the caveat that differences between solution and solid-phase dynamics may complicate this picture. MD simulations of XL177A binding with covalent labeling of C223 and H-bonding to Q297 taken as priors showed a decrease in water contacts of the α4/5 and S4-S5 residues as well as an increase in water contacts for the region from α2 to α4 when compared to the unbound USP7 construct, consistent with HDX experimental data. Additionally, final snapshots after 150 ns production stages show a binding mode similar to that of the XL188-USP7 construct (Fig. S7). Taken together, these data suggest that XL177A possesses similar binding sites and a similar binding mode to that of XL188 but induces additional conformation changes in protein dynamics compared to XL188. Similar effects were observed when full length USP7 was bound to XL177A (data not shown).

### XL177A is a highly selective USP7 chemical probe

**Figure 4:**
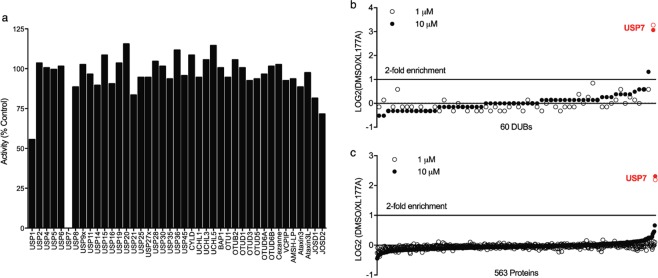
XL177A is selective for USP7. (**a**) Remaining activity of 41 purified recombinant DUBs against Ub-Rho after 15-minute pre-treatment with XL177A (n = 1). (**b**) Ratio of Bio-Ub-PA/VME labeling for 60 DUBs in HEK293AD lysate between samples pre-treated for 5 hours with DMSO v. 1 μM XL177A (n = 2 experimental replicates with two technical replicates, reported values are medians of all replicates). **(c**) Ratio of XL177A-DTB labeling for 566 proteins in HEK293AD lysate between samples pre-treated for 5 hours with DMSO v. 1 μM XL177A (n = 3 experimental replicates with two technical replicates, reported values are medians of all replicates).

The selectivity of XL177A was first assessed by determining its inhibitory activity across a panel of 41 recombinant DUBs using *in vitro* activity assays. At a concentration of 1 µM (>1000-fold higher than its IC_50_ for USP7), XL177A completely inhibited USP7 enzymatic activity but did not exhibit significant activity against any other DUBs (Fig. 4a). The DUB enzymes in this panel primarily consist of only domains or binding partners that are sufficient for *in vitro* activity, and many DUBs are large multi-domain proteins and/or exist in macromolecular complexes. Furthermore, the standard conditions for this panel include compound pre-incubations of 15 minutes, limiting our ability to assess off-targets that are inhibited with time-dependent kinetics. We thus utilized competitive ABPP with quantitative MS to explore the selectivity of XL177A in a more native context. Briefly, either DMSO or XL177A was pre-incubated with HEK293 crude cell extract for 5 hours. The lysate was then incubated with a 1:1 mixture of biotin-ubiquitin-propargylic acid (Bio-Ub-PA) and biotin-ubiquitin-vinyl methyl ester (Bio-Ub-VME), an ABP combination that maximized DUB biotin labeling in our hands (Fig. S8). The labeled lysates were enriched by streptavidin resin, tandem mass tag (TMT)-labeled, combined and analyzed by LC/MS. We found that XL177A significantly blocked USP7 labeling by DUB ABPs in a dose-dependent manner while remaining selective against 59 other DUBs (Fig. 4b). Collectively, using state-of-the-art DUB *in vitro* and activity-based proteomic profiling, we demonstrated that XL177A binds to and inhibits USP7 with >10-fold selectivity over closely related DUBs.

To define XL177A specificity proteome-wide, we assessed binding partners using an unbiased chemical proteomics screen. First, we synthesized XL177A-DTB, an XL177A analog with a desthiobiotin (DTB) affinity tag, and demonstrated that it retained USP7 inhibitory activity (Table S1). Streptavidin affinity pulldowns of XL177A-DTB followed by on-bead digest and proteomic analysis identified 566 proteins including USP7. Since this approach not only identifies targets of XL177A but also endogenously biotinylated or highly abundant native proteins, we performed a competition experiment with XL177A to identify proteins that were bona fide targets of XL177A. Specifically, we treated HEK293 cell lysates with XL177A for 5 hours at 1 or 10 μM, incubated with XL177A-DTB, and quantified concentration-dependent blocking of XL177A-DTB binding throughout the proteome. Of the 566 proteins covalently modified by XL177A-DTB, only USP7 exhibited>3-fold inhibition of labeling when treated with XL177A at 1 or 10 μM (Fig. 4c). These findings corroborate that XL177A is highly specific for USP7 relative to other DUBs and further, the rest of the proteome. The tetrahydroacridine warhead of XL177A is closely related to the biologically quiescent cysteine electrophile 4-chloro-pyridine^32^, which may help to explain XL177A’s selectivity.

Taken together, these data demonstrate that XL177A possesses cellular permeability, highly potent target engagement with native USP7, and exquisite proteome-wide selectivity for USP7. This compound, in combination with XL177B, meets the criteria of potency, selectivity, and cellular activity set out by multiple organizations to describe well-characterized chemical probes^33,34^. We thus sought to use XL177A/B to gain a deeper understanding of the role of USP7 in cancer using unbiased profiling experiments.

### Transcriptional profiling of XL177A reveals strong enrichment of p53 target genes

**Figure 5:**
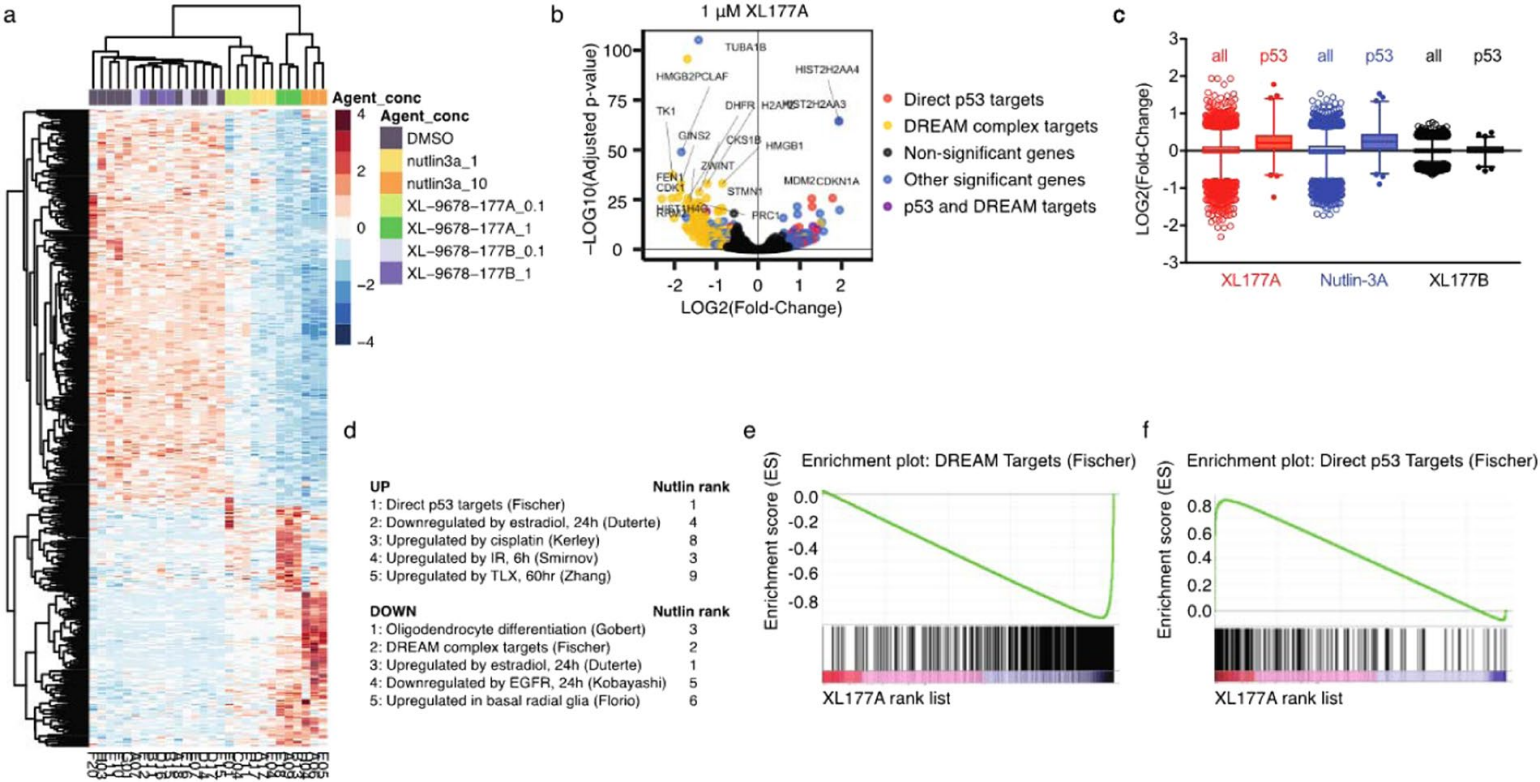
XL177A activates p53 signaling transcriptome-wide. (**a**) Heat map showing hierarchical clustering of transcriptional profiles from MCF7 cells treated for 24 hours with 0.1 or 1 μM XL177A, 0.1 or 1 μM XL177B, or 1 or 10 μM Nutlin-3A. (**b**) Volcano plots of genes enriched or depleted after 24-hr treatment with 1 μM XL177A. (**c**) Expression levels of all genes (clear) or direct p53 target genes (filled) from MCF7 cells treated for 24 hours with 1 μM XL177A (adjusted p < 0.0001), Nutlin-3A (adjusted p < 0.0001), or XL177B (ns). Box: inter-quartile range; Whiskers: gens within 3 SD of mean. (**d**) Top 5 datasets enriched in the set of genes significantly up- or down-regulated by 1 μM XL177A treatment and their ranking in the set of genes up- or down-regulated by Nutlin-3A. (**e,f**) Waterfall plot of all detected transcripts from 1 μM XL177A ranked by normalized expression score and compared to DREAM complex targets (**e**) or direct p53 targets (**f**).

USP7 directly and indirectly regulates protein levels and subcellular localization of multiple transcription factors and epigenetic modifiers including p53^35^, N-Myc^21^, FOXO4^20^, DNMT1^36^, EPOP^37^, and PRC1^38^. In order to systematically evaluate the cellular effects of USP7 inhibition in an unbiased manner, we first evaluated the transcriptome-wide effects of XL177A and XL177B in MCF7 cells. We tested the MDM2/p53 interaction inhibitor Nutlin-3A in parallel to benchmark USP7 inhibition against a validated MDM2 inhibitor. To this end, we treated MCF7 cells for 24 hours with low or high doses of XL177A, XL177B, or Nutlin-3A, then used high-throughput 3’ Digital Gene Expression (DGE) RNA-seq to analyze the transcriptome-wide effects of these compounds^39,40^. Overall, we detected transcripts for 7,000–10,000 genes (7276 genes detected across all conditions). Interestingly, hierarchical clustering revealed that XL177A and Nutlin-3A transcriptional profiles were correlated at both low (0.1 μM XL177A and 1 μM Nutlin-3A) and high (1 μM XL177A and 10 μM Nutlin-3A) doses, while XL177B clustered with the vehicle (DMSO)-treated control cells (Fig. 5a). While XL177A did upregulate a set of genes not affected by Nutlin-3A, the overall transcriptional profile of these compounds led us to systematically evaluate whether they were enriched for similar gene sets.

To identify specific gene sets that were enriched under different treatment conditions, we computed the overlap between significantly up- or down-regulated genes and the gene sets in the Molecular Signatures Database^41^. A recent meta-analysis of p53 target genes had been generated in part by RNA-seq data from Nutlin-3A-treated MCF7 cells^16^, and we found that the upregulated genes from both low- and high-dose Nutlin-3A were enriched for the direct p53 target genes from this meta-analysis. The same report had also identified a set of dimerization partner, Retinoblastoma-like, E2F and multi-vulval class B (DREAM) complex target genes by combining p53 repression data (generated in part with Nutlin-3A-treated MCF7 cells) with cell cycle expression and DREAM component binding. Again, we found that this DREAM target geneset was most strongly enriched in the downregulated genes from both Nutlin-3A treatments. Interestingly, we also found that both low- and high-dose XL177A strongly upregulated the p53 targets (most significant for both doses) and strongly downregulated the DREAM complex targets (most significant for high dose and second most significant for low dose) (Fig. 5b–f).

USP7 inhibition by XL177A thus elicits a transcriptional signature that strongly phenocopies that of direct MDM2 inhibition by Nutlin-3A, suggesting that p53 upregulation may be the most relevant consequence of USP7 inhibition in *TP53*-WT MCF7 cells. These results were somewhat surprising, as we expected to identify multiple gene signatures relevant to other USP7 targets. We considered that XL177A may produce different effects in *TP53*-WT versus *TP53*-mutant cells and thus sought to profile XL177A in a panel of cancer cell lines with diverse genomic backgrounds.

### Selective USP7 inhibition is correlated to *TP53* mutational status in a diverse panel of cell lines

**Figure 6:**
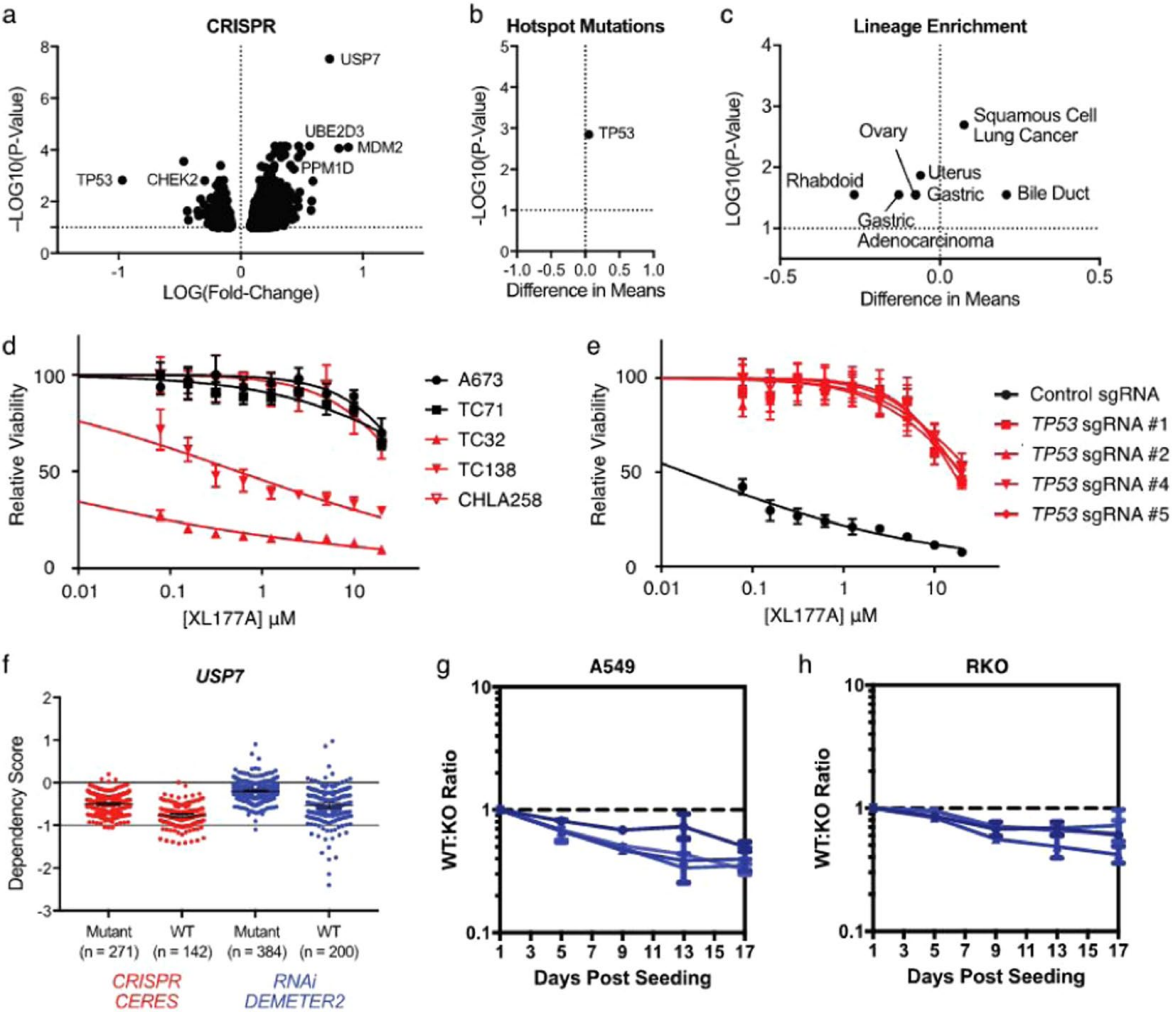
XL177A acts through a p53-dependent mechanism. (**a**) Correlation between relative proliferation profiles of 484 cancer cell lines after XL177A treatment for 5 days or CRISPR KO with the indicated sgRNA. (**b**) Depmap was queried to determine whether mutations in any of >18,000 genes correlated with XL177A AUC. Only *TP53* mutant cell lines displayed significantly altered response relative to WT. (**c**) Lineages enriched as sensitive (left of 0) or resistant (right of 0) to XL177A in the PRISM data set. (**d**) Relative Cell Titer Glo luminescence of a panel of *TP53*-WT (red) or *TP53*-mutant (black) Ewing Sarcoma cell lines after treatment with XL177A for 3 days. (**e**) Relative Cell Titer Glo luminescence of TC32 cells expressing the indicated sgRNA after treatment with XL177A for 3 days. (**f**) Correlation between USP7 DEMETER and AVANA dependency scores in the Broad Depmap portal for *TP53*-WT (red) and *TP53*-mutant (black) cell lines. (**g**) Log_10_ ratio of A549-FF to A549-sg*TP53*-Renilla cells after treatment of an initial 1:1 mixture of the two cell lines with the indicated sgRNA for the indicated number of days.

We screened XL177A, XL177B, and Nutlin-3A in 8-point dose response against a panel of 484 cancer cell lines using barcoded cancer cell lines/PRISM technology^42^. Briefly, cell lines in average pool size of 25 were treated with inhibitor for 5 days, then the relative abundance of each cell line was determined by high-throughput sequencing. Calculated area under curve (AUC) for each cell line was determined and utilized to determine sensitivity. Correlation of viability profiles between XL177A and XL177B was only observed at the two highest doses of XL177B, which is consistent with an on-target effect overall (Fig. S9). The viability profile observed for XL177A was compared to the viability profiles in the genetic knockout profiles from the DepMap cancer dependency map (Fig. 6a)^43^. Sensitivity to XL177A correlated most highly with USP7 KO, supporting the conclusion that XL177A’s effects are on-target. Interestingly, XL177A sensitivity was highly correlated with *MDM2* and *UBE2D3* (an E2 conjugating enzyme for p53) KO and highly anti-correlated with *TP53* KO, implying that p53 is a key predictor of response to USP7 inhibition across the entire panel of cells. In addition to genetic modulation, features that correlate with viability response to XL177A were interrogated using publicly available gene expression, copy number, and mutation profiles from the DepMap portal. Notably, a mutation in *TP53* predicted decreased sensitivity to XL177A (∆ = 0.0512, p = 0.0014) (Fig. 6b). The strength of association was similar to that of Nutlin-3A (∆ = 0.0919, p < 0.0001) (Fig. S10). No other mutations were associated with the sensitivity profile of XL177A, and *TP53* mutations were not significantly associated with a differential response to XL177B. Taken together, these data suggest that the mechanism of action of XL177A is USP7 inhibition and that sensitivity to USP7 inhibition is highly associated with *TP53* mutational status.

### Selective USP7 inhibition exerts p53-dependent growth suppression in individual cell lines

To validate our results from the unbiased screen, we tested effects of XL177A, XL177B and Nutlin-3A using individual cell lines. We chose to prioritize studies in Ewing Sarcoma^30^, acute myeloid leukemia (AML)^26^, and multiple myeloma (MM)^24^ because these lineages have been evaluated with previous generations of USP7 inhibitors. As rhabdoid tumors were identified as the lineage most sensitive to both XL177A and Nutlin-3A in the cell profiling experiment we performed (Fig. 6c), we also included rhabdoid cell lines in our testing. We assessed the effect of XL177A in panels of these four cell lineages and found that *TP53* functional status predicted response to USP7 inhibition: 9 out of 10 *TP53*-WT cell lines were XL177A-sensitive, compared to only 1 out of 17 *TP53*-mutant cell lines (Figs. 6d and S11). XL177B was ~100-fold less potent in the cell lines tested, consistent with the effects being USP7-dependent (Figs. S11 and S12).

We had previously reported a series of stable *TP53*-KO TC32 cell lines^30^, and given the strong growth suppression of TC32 by XL177A, we assessed its effect in the matched *TP53*-KO cell lines. Interestingly, we found that *TP53* KO completely rescued TC32 growth except at high doses, indicating that this effect is p53-dependent (Fig. 6e). Meanwhile, GNE-6640, a USP7 inhibitor that does not exhibit global p53-dependent effects^26^, showed little selectivity for *TP53*-WT Ewing Sarcoma lines or *TP53*-WT TC32 cells versus *TP53*-mutant or *TP53*-KO cells (Fig. S12), a result that parallels our previous findings with the USP7 inhibitor P5091^30^. These results suggest that, while the globally selective USP7 inhibitor XL177A acts through a p53-dependent mechanism, P5091 and GNE-6640 may have as yet unknown targets that lead to p53-independent effects.

### Genetic studies validate *TP53* status as a predictor of response to USP7

Given the highly p53-dependent nature of response to XL177A, we sought to identify whether genetic modulation of USP7 also produces p53-dependent effects in cancer cell lines. We first queried the DepMap portal^43^, which houses genome-scale loss-of-function datasets interrogating gene essentiality across hundreds of genomically characterized cancer cell lines. Both RNAi (DEMETER2, n = 711) and CRISPR-Cas9 (CERES, n = 436) targeting *USP7* produced datasets that were strongly correlated with a number of p53-network genes, including *MDM2* (RNAi R^2^ = 0.598; CRISPR R^2^ = 0.575), *MDM4* (DEMETER2 R^2^ = 0.522; CERES R^2^ = 0.356) and *PPM1D* (DEMETER2 R^2^ = 0.346; CERES R^2^ = 0.515), *TP53* (DEMETER2 R^2^ = −0.534; CERES R^2^ = −0.496), and *CHEK2* (DEMETER2 R^2^ = −0.372; CERES R^2^ = −0.406) (Table S3). In order to specifically assess the role of p53 in response to genetic modulation of *USP7*, we annotated individual cell lines as *TP53*-functional-WT or functional-mutant using the method in Giacomelli et al^44^. and performed two-way T-tests comparing the WT and mutant populations. In both the DEMETER2 (mean diff.=0.344 ± 0.027, p < 0.0001, n = 584) and CERES (mean diff.=−0.273 ± 0.025, p < 0.0001, n = 413), *TP53*-functional status was significantly correlated to response to *USP7* genetic modulation (Fig. 6f). Overall, available genetic data confirm that response to *USP7* modulation is correlated with *TP53* status across multiple cancer cell lines.

To further assess the functional role of p53 on USP7 modulation in *TP53*-WT cell lines, we employed a dual luciferase reporter assay to assess the comparative fitness of *TP53*-WT and *TP53*-KO cells in response to *USP7* KO^44^. Briefly, we expressed Firefly (FF) luciferase in parental A549 and RKO cells and Renilla luciferase in stable *TP53*-KO A549 and RKO cells. The FF- and Renilla-expressing cells were mixed in a 1:1 ratio and then exposed to Cas9 and sgRNAs targeting *MDM2*, *USP7*, *TP53*, *CDKN1A*, LacZ, or FF luciferase for 17 days. Both sg*MDM2* and sg*USP7* led to sustained reductions in the parental cells of both A549 and RKO (Figs. 6G and S13), indicating that *TP53*-KO improves the fitness of these cells in response to USP7 or MDM2 modulation. Thus, the cell killing effect of *USP7* KO is, as with *MDM2* KO and XL177A treatment, at least partially mediated by p53 in *TP53*-WT cells.

## Discussion

DUBs are an emerging class of potential drug targets. Multiple lines of evidence have led to the classification of numerous DUBs as drivers of disease across diverse therapeutic areas. In oncology, many DUBs have been classified as oncogenic based on their regulatory role on substrate proteins involved in tumorigenesis^45^. Until recently, the excitement originating from these studies has been counterbalanced by uncertainty regarding the targetability of DUBs. However, recent studies describing development of highly potent and selective inhibitors of DUBs, including USP7 and CSN5^46^, have led to a turning point in the field, re-energizing interest in DUBs as tractable targets for drug discovery. The prevailing current approach to DUB inhibitor development is based on designing non-covalent, allosteric inhibitors. Here, we approach DUB inhibitor development from a different angle and report the first highly potent (sub-nanomolar) and selective (proteome-wide) irreversible DUB inhibitor. The compound, XL177A, inhibits USP7 with an IC_50_ of 0.34 nM and binds and inhibits native USP7 with potencies in the low nanomolar range. Notably, proteome-wide chemoproteomic experiments revealed exquisite selectivity for USP7, with no proteins other than USP7 being substantially labeled by XL177A. Consistent with this high degree of selectivity, several lines of evidence strongly support the notion that phenotypes observed with XL177A treatment are on-target. First, we paired XL177A with its inactive enantiomer (XL177B), in a wide range of signaling and proliferation experiments and observe loss of phenotype with XL177B. Moreover, comparison of the sensitivity profile of XL177A across over 400 cancer cell lines with sensitivity profiles generated in those same cell lines with KD / KO of over 18,000 genes identified *USP7* KO as exhibiting the most similar profile. The combination of potency, proteome-wide selectivity, and evidence of on-target mechanism across large panels of cancer cell lines nominate XL177A as a best-in-class cellular probe of USP7. We note that XL177A is not an *in vivo* probe, and we thus have a limited understanding of its selectivity, potency, and potential toxicity in more complex animal models. Nonetheless, the cellular properties of XL177A make it a highly valuable tool for the research community in unraveling USP7 biology and addressing fundamental questions surrounding DUB function more broadly.

Using XL177A, paired with XL177B, we focused our investigations on USP7 substrates or cellular features that predict growth suppressive effects in cancer in response to pharmacological inhibition of USP7. Overwhelmingly, our studies revealed mutational status of the tumor suppressor p53 to be the key cellular feature predicting sensitivity to USP7 by XL177A. Out of more than 18,000 genes analyzed, only mutations in *TP53* significantly correlated with response to XL177A across a panel of more than 400 cell lines. Tested individually, 9 out of 10 *TP53*-WT cell lines were XL177A-sensitive, compared to only 1 out of 17 *TP53*-mutant cell lines. In addition, *TP53* KO rendered *TP53*-WT cells resistant to XL177A, and transcriptome-wide response to XL177A phenocopied effects of Nutlin-3A, a validated inhibitor of MDM2-mediated p53 ubiquitination and therefore a compound expected to stabilize p53 and inhibit its proteasomal degradation.

*TP53* is one of the most commonly mutated genes in cancer. Due to its role in regulating cell cycle arrest and apoptosis, its suppression is a critical node in cancer progression, and p53 re-activation is a key therapeutic strategy across all cancer types. Despite the high *TP53* mutation rate in cancer, several cancer types have a low rate of *TP53* mutation, especially hematopoietic and pediatric malignancies. For these *TP53*-WT cancers, p53 activation can be achieved using direct p53 stabilizers such as MDM2 inhibitors, MDMX inhibitors, and Wip1 inhibitors. These p53 activating agents act synergistically with each other and with DNA-damaging radiation and chemotherapy in *TP53*-WT tumors, leading to clinical trials that combine p53 activators and cytotoxic drugs in multiple myeloma, acute myeloid leukemia, and B cell lymphoma. However, clinical trials with MDM2 inhibitors have already revealed acquired resistance, and additional pathways that can be modulated to activate p53 are of great interest for the treatment of *TP53*-WT tumors. We previously reported that the USP7 inhibitor XL188 acted as a p53 activator in *TP53*-WT Ewing sarcoma, but studies of USP7 inhibition in other contexts have typically focused on p53-independent mechanisms. Here, we establish that the p53-dependent activity of USP7 inhibition is of great importance for growth suppression of cancer cells across all lineages. We propose that USP7 inhibitors are p53 activators that should be explored clinically as single agents or in combination studies in hematopoietic and pediatric malignancies with a low rate of *TP53* mutation. While USP7 inhibition may stabilize p53 by inducing MDM2 degradation, we note that the p53-dependent effects of USP7 inhibition may not be solely driven by MDM2. Previous studies have shown that USP7 inhibition or knockdown also leads to decreased DNA damage tolerance via degradation of RAD18 (replication-associated repair)^47^, CSB (nucleotide excision repair)^48^, and ALKBH2/3 (alkylation repair)^49^, and USP7 has been shown to regulate both p14/Arf (which mediates p53 stability) and Tip60 (which mediates p53 transcriptional activity)^50,51^. Further insights into these USP7 substrates may furnish a better understanding of the molecular mechanism of the p53 dependence we observe for our inhibitors.

In conclusion, here we report the first highly potent (sub-nanomolar) and selective (proteome-wide) irreversible DUB inhibitor. Our results demonstrate that covalent targeting of the active site of DUBs is a viable strategy for probe and drug discovery and further, support exploration of a target-class approach to rapidly discover covalent hits, leads and probes for multiple DUBs. Moreover, using the newly developed selective USP7 inhibitor we now provide an answer to a long-standing question surrounding the USP7/p53 regulator axis and demonstrate that *TP53* status determines the response to selective USP7 inhibition, with *TP53*-WT cell lines found to be sensitive. Together, the probe we developed and the insights into the p53 dependency of its activity provide valuable information for further advancement of both basic biology and clinical translation of USP7.

## Methods

### Antibodies, cell lines, and reagents

HDM2 (sc-965) antibody was obtained from Santa Cruz. p53 (9282s), p21 (2947s), GAPDH (2118s), and USP7 (4833s) antibodies were obtained from Cell Signaling Technology. Ub-AMC (U-550) and HA-Ub-VS (U-212) were obtained from Boston Biochem. Bio-Ub-PA (UbiQ-076) and Bio-Ub-VME (UbiQ-054) were obtained from UbiQ Bio. BAX (Hs00180269_m1), CDKN1A (Hs00355782_m1), DDB2 (Hs03044953_m1), GADD45A (Hs00169255_m1), GAPDH (402869), MDM2 (Hs00540450_s1), and TP53 (Hs01034249_m1) Taqman probes were obtained from Thermo-Fisher. MCF7 cells were a generous gift from Jean Zhao’s laboratory, and NCI-H1975 cells were a generous gift from Pasi Jänne’s laboratory. HEK 293AD, G401, G402, and MESSA were purchased from ATCC. Stable *TP53* knockout TC32 and A549 cells were previously described in Stolte *et al*. and Giacomelli *et al*., respectively^30,44^.

### *USP7* Cloning, expression, and purification

The constructs encoding *USP7* full length (amino acids 1–1102) and catalytic domain (208–560) used were cloned as described^29^. Both constructs were overexpressed in E. coli BL21 (DE3). Cells were grown at 37 °C to an OD of 0.9, cooled to 16 °C, induced with 500 μM isopropyl-1-thio-D-galactopyranoside (IPTG), incubated overnight at 16 °C, collected by centrifugation, and stored at −80 °C. Cell pellets were sonicated in lysis buffer (25 mM Tris pH 8, 1 M NaCl, and 10 mM BME) supplemented with 10 μg/ml phenylmethanesulfonylfluoride (PMSF) and the resulting lysate was centrifuged at 30,000 xg for 40 min. Ni-NTA beads (Qiagen) were mixed with lysate supernatant for 2 hours, and washed with lysis buffer supplemented with 25 mM imidazole. The bound protein was eluted with lysis buffer supplemented with 300 mM imidazole. The sample was then concentrated to 1 ml (30 kDa concentrator; Amicon Ultra, Millipore), and run on a Superdex 200 (GE healthcare) in buffer containing 25 mM HEPES pH 7.5, 200 mM NaCl, and 1 mM DTT. Fractions were pooled, concentrated and frozen at −80 °C. The construct encoding the catalytic domain of USP7 C223A was generated by site-directed mutagenesis, and the protein was purified as described for the WT enzyme.

### Ub-AMC Enzymatic and kinetic assays

Full length USP7 was tested for its activity in Ubiquitin-AMC assay in presence or absence of inhibitors. USP7 (5 nM) was pre-incubated for 6 hours at room temperature with different concentrations of inhibitors or DMSO as a control in 50 mM HEPES pH 7.5, 0.5 mM EDTA, 11 uM ovalbumin, and 5 mM DTT. Ubiquitin-AMC (Boston Biochem) was then added to a final concentration of 500 nM. The initial rate of the reaction was measured by collecting fluorescence data at one minute intervals over 30-minute period using a Clariostar fluorescence plate reader at excitation and emission wavelength of 345 and 445 nm respectively. The calculated initial rate values were plotted against inhibitor concentrations to determine IC_50_s. All the experimental data were plotted using Prism GraphPad. All assays for each compound were performed at least twice for each compound.

To calculate the *k*_*i*_ and *k*_*ianct*_ values for XL-9678-041, the procedure above was used, but different concentrations of inhibitor were incubated with USP7 for different time points (5 min - 3 hours) before adding Ubiquitin-AMC. To determine *k*_*obs*_, the time course curves were fit to the equation y  =  y_max_(1 − exp(−*k*_*obs*_⋅x)). The *k*_*obs*_ values were then plotted against the inhibitor concentrations and fit to the equation y = *k*_*inact*_/(1 + (*k*_*i*_/x)) to obtain the values for *k*_*i*_ and *k*_*ianct*_.

### MS Labeling

Purified USP7 catalytic domain was diluted to 20 μM in 10 μL labeling buffer (20 mM HEPES pH 7.5, 150 mM NaCl, 1 mM TCEP) and incubated for the indicated times with 50 μM (2.5×) compound. After incubation, samples were flash frozen in liquid nitrogen and stored at −80 °C until analysis.

#### Intact MS Analysis

Intact mass analysis was performed by injecting 5 µg USP7 catalytic domain onto a self-packed reversed phase column (1/32” O.D. × 500 um I.D., 5 cm of POROS 10R2 resin). After desalting, protein was eluted with an HPLC gradient (0–100% B in 4 minutes, A = 0.2 M acetic acid in water, B = 0.2 M acetic acid in acetonitrile, flow rate ~30 µL/min) into an LTQ ion trap mass spectrometer (ThermoFisher Scientific, San Jose, CA). Mass spectra were processed using MagTran1.03b2^52^.

#### CE-MS Analysis

To identify sites of covalent modification, treated protein was reduced (10 mM dithiothreitol), alkylated (22.5 mM iodoacetamide), and digested with trypsin overnight at 37 °C. Peptides were desalted using SP3^53^, dried by vacuum centrifugation, and reconstituted in 1% formic acid/50% acetonitrile with 100 mM ammonium acetate. Peptides were then analyzed by CE-MS using a ZipChip CE system and autosampler (908 Devices, Boston, MA) interfaced to a QExactive HF mass spectrometer (ThermoFisher Scientific, San Jose, CA). Peptide solution was loaded for 30 seconds, and the mass spectrometer was operated in data dependent mode and subjected the 5 most abundant ions in each MS scan (60k resolution, 3E6 target, lock mass enabled) to MS/MS (15k resolution, 1E5 target, 100 ms max inject time). Dynamic exclusion was enabled with a repeat count of 1 and an exclusion time of 6 seconds. MS/MS data was extracted to.mgf using mulitplierz scripts^54,55^ and searched against a forward-reverse human NCBI refseq database using Mascot version 2.6. Search parameters specified fixed carbamidomethylation of cysteine, and variable oxidation (methionine) and XL177A modification (cysteine). Precursor mass tolerance was set to 10 ppm and product ion tolerance was 25 mmu. Spectral validation was performed using mzStudio^56^.

### Cell culture

MCF7 cells were cultured in RPMI-1640 growth medium supplemented with 10% FBS. HEK293AD cells were cultured in DMEM + 10% FBS + 1% penicillin-streptomycin. A673 cells were cultured in DMEM + 10% FBS + 1 mM sodium pyruvate + 1%PSQ. TC32 and TC71 cells were cultured in RPMI + 10% FBS + 1% PSQ. TC138 and CHLA258 cells were cultured in IMDM + 20% Fetal Bovine Serum + 4mM L-Glutamine + 1X ITS (5 µg/mL insulin, 5 µg/mL transferrin, 5 ng/mL selenous acid). G401, G402, and MES-SA cells were cultured in McCoy’s Modified Media (ATCC) + 10% FBS. SKNO-1-luc + , SKM-1, NB4-luc + , HL60, HEL, MV4,11, OCI-AML3, and MOLM14 cells were cultured in RPMI + 10% FBS + 2% L-glutamine + 1% penicillin-streptomycin. All cell lines were maintained in 10 cm^2^ tissue-culture treated dishes 37 °C in a 5% CO_2_ incubator. All cell lines were verified Mycoplasma-free by the MycoAlert test kit.

### HA-Ub-VS Labeling

HA-Ub-VS experiments were performed as previously described in *Lamberto et al*^29^. Briefly, target engagement lysis buffer (50 mM Tris pH 8.0, 150 mM NaCl, 5 mM MgCl_2_, 0.5 mM EDTA, 0.5% NP-40, 10% glycerol, 1 mM TCEP, protease and phosphatase inhibitors) was added to cell pellets on ice. Lysate was cleared by centrifugation and diluted to 1.67 mg/mL. Where indicated, 30 μL lysate was then incubated with inhibitors or DMSO for the indicated timepoints. 1 μM HA-Ub-VS was then added to the lysate and incubated at RT for the indicated time points. Labeling reactions were quenched with 4x LDS sample buffer (Thermo Fisher B0007) supplemented with 10% BME, vortexed vigorously, and heated to 95 C for 5 minutes. Samples were resolved by SDS-PAGE and analyzed by Western blot with the indicated antibodies.

### Real-Time PCR

After cell treatment under the indicated conditions, total cellular RNA was purified using a Qiagen RNEasy kit. 1 μg of RNA was then converted to cDNA using SuperScript III First-Strand Synthesis (Invitrogen). cDNA from each sample was then combined with the indicated TaqMan probe and 2x MasterMix in a 96-well Fast RT-PCR plate (Invitrogen). qPCR was performed on an Invitrogen 7500 Fast qPCR instrument and gene expression was calculated using the 2^-∆∆Ct^ method on Graphpad Prism.

### Cell cycle analysis

For propidium iodide (PI) staining, treated cells (~1 million per condition) were washed with cold PBS, then fixed in 80% ethanol overnight at −20 °C. After fixing, cells were pelleted, washed with PBS, and reconstituted in 500 μL FxCycle PI / RNAse A staining solution (Thermo Fisher). Cells were stored overnight at 4 °C and analyzed using a BD Fortessa flow cytometer.

### Hydrogen deuterium exchange

Experiments were performed as previously described^29^. Briefly, USP7 (50 µM in 20 mM Hepes (pH 7.5), 200 mM NaCl, 1 mM TCEP, 5% glycerol H_2_O) was pre-incubated at room temperature (21 °C) with the indicated compound in a 10:1 compound:USP7 ratio (XL041 for 60 min, XL177A for 30 min), then diluted with 15-fold D_2_O buffer (pD 7.5) at room temperature. At each deuterium exchange time point (from 10 s to 4 hours) an aliquot from the exchange reaction was removed and acidified (pH to 2.5) with an equal volume of quench buffer (0.8% formic acid and 0.8 M guanidine hydrochloride, H_2_O) to quench the reaction. Quenched samples were immediately injected into the LC/MS system. Samples were digested online using a Poroszyme immobilized pepsin cartridge (2.1 mm × 30 mm, Applied Biosystems) at 15 °C for 30 s, then injected into a custom Waters nanoACQUITY UPLC HDX Manager and analyzed on a XEVO G2 mass spectrometer (Waters Corp., USA). Peptides were compared to highly deuterated peptide standards to determine the average amount of back-exchange (~20–30%). Deuterium levels were not corrected for back-exchange and are therefore reported as relative^31^. All experiments were performed in duplicate, and the error of measuring the mass of each peptide averaged ± 0.15 Da. The HDX MS data were processed using PLGS 3.0 and DynamX 3.0 (Waters Corp., USA). Common peptides that were compared between the USP7 catalytic domain alone and bound to the compounds led to a sequence coverage of 85.4% with 81 peptic peptides for XL-041 and 94% with 105 peptic peptides for XL-177A (Supplemental Fig. S7A,B).

### Molecular dynamics simulations

The models of the XL188-bound USP7 complex were constructed from the co-crystalized PDB crystal structure (PDBID: 5VS6). The model of the XL177A- and XL177B-bound USP7 complexes were constructed from the apo and Ub-Aldehyde bound USP7 crystal structures (PDBIDs: 1NB8 and 1NBF, respectively). With the preparation and simulation protocols tested in our prior studies^57–59^, all protein structures were prepared using Protein Preparation Wizard, solvated in SPC water with sodium counter ions by the System Builder, and simulated with the NPT ensemble (300 K, 1 atm, Martyna-Tuckerman-Klein coupling scheme) using the OPLS3e force field^60^ in Desmond v5.4^61^. Each construct underwent minimization, equilibration, and 150-ns production stages with a time step of 2 fs. The Ewald technique with a 9 Å cutoff was used for the Van der Waals and other electrostatic calculations. Hydrogen atoms were constrained using the SHAKE algorithm. At least two replicas were collected for each construct. Trajectory visualization and data analysis was done with the VMD program^62^ and in-house TCL and Python scripts.

### *In Vitro* DUB Profiling

Compounds were screened using the Ubiquigent Drug Profiler SPT system (www.ubiquigent.com/drug-discovery-services/dubprofiler/). Each of 41 purified DUBs was incubated with compound for 15 minutes, then ubiquitin rhodamine 110 (Ub-Rho) was added and percent inhibition determined based on fluorescence relative to a DMSO control.

### *In Situ* DUB Profiling

DUB profiling was performed using conditions similar to those in Lawson et al^63^. HEK 293AD cells were lysed using target engagement lysis buffer (50 mM Tris pH 8.0, 150 mM NaCl, 5 mM MgCl_2_, 0.5 mM EDTA, 0.5% NP-40, 10% glycerol, 1 mM TCEP, protease and phosphatase inhibitors), and the lysate was cleared by centrifugation. Samples were diluted to 2 mg/mL, and 1 mL lysate was incubated with the indicated concentration of XL177A for 5 hours at RT. Excess inhibitor was removed using a 30 K Amicon spin filter, then the resulting lysate was incubated with 1 μM each of Biotin-Ub-PA and Biotin-Ub-VME for 90 minutes at RT. SDS was added to a final concentration of 1.2%, and samples were heated to 80 °C for 5 minutes. After cooling to RT, 1X PBS was added to dilute the final SDS concentration to 0.2%. 100 μL streptavidin agarose slurry was added to each sample, followed by incubation at RT for 3 hours. After streptavidin enrichment, samples were washed vigorously (2×0.2% SDS, 3x PBS, 3x ddH_2_O). After the final wash, all supernatant was removed using a flat-bottom tip, and the resin was flash frozen and stored at −80 °C until workup for TMT labeling.

### XL177A-DTB Profiling

HEK 293AD cells were lysed as described above, and the lysate was cleared by centrifugation. Samples were diluted to 10 mg/mL, and 200 μL lysate (2 mg protein total) was incubated with the indicated concentrations of XL177A for 4 hours at RT, then 2 μM of WH114A for 4 additional hours. SDS was added to a final concentration of 1.2% (27.2 μL of a 10% stock), and denatured by heating to 80 °C for 5 minutes. After cooling to RT, 1125 μL 1X PBS was added to dilute the final SDS concentration to 0.2%. 50 μL streptavidin agarose slurry was added to each sample, followed by incubation at RT for 3 hours. After streptavidin enrichment, samples were washed vigorously (2×0.2% SDS, 3x PBS, 3x ddH_2_O). After the final wash, all supernatant was removed using a flat-bottom tip, and the resin was flash frozen and stored at −80 °C until workup for TMT labeling.

#### Sample prep for mass spectrometry analysis

Streptavidin beads were resuspended in 100 mM Tris pH 8.0 and bound proteins were denatured with 0.1% rapigest, reduced (10 mM dithiothreitol), alkylated (22.5 mM iodoacetamide), and digested with trypsin overnight at 37 °C. To remove rapigest, recovered supernatants were acidified with 10% TFA, incubated at 37^o^ C for 45 minutes, and centrifuged at 14,000 rpm for 15 minutes at 4^o^ C. Peptides were then desalted by C18, and dried by vacuum centrifugation. Dried peptides were reconstituted in 50 mM pH8.0 TEAB and labeled with TMT reagent at RT for 1 hour. TMT reactions were pooled and treated with hydroxylamine according to the manufacturer’s instructions. Peptide mixtures were then dried, reconstituted in 50 mM ammonium bicarbonate and desalted by SP3^53^. Eluted peptides were then analyzed by nanoflow LC-MS/MS as described^64^ with a NanoAcquity UPLC system (Waters, Milford, MA) interfaced to a QExactive HF mass spectrometer (Thermofisher Scientific, San Jose, CA). TMT labeled peptides were injected onto a precolumn (4 cm POROS 10R2, Applied Biosystems, Framingham, MA), resolved on an analytical column (30 µm I.D. × 50 cm packed with 5 µm Monitor C18) and introduced to the mass spectrometer by ESI (spray voltage = 3.5 kV, flow rate ~30 nL/min). The mass spectrometer was operated in data dependent mode such that the 15 most abundant ions in each MS scan (*m/z* 300–2000, 120 K resolution, target = 3E6, lock mass for 445.120025 enabled) were subjected to MS/MS (m/z 100–2000, 30 K resolution, target = 1E5, max fill time = 100 ms). Dynamic exclusion was selected with a repeat count of 1 and an exclusion time of 30 seconds. MS/MS data was extracted to.mgf using mulitplierz scripts^54,55^ and searched against a forward-reverse human NCBI refseq database using Mascot version 2.6. Search parameters specified fixed carbamidomethylation of cysteine, fixed N-terminal and lysine TMT labelling, and variable oxidation (methionine). Additional multiplierz scripts were used to filter results to 1% FDR and derive protein-level aggregate reporter ion intensities using peptides mapping uniquely into the genome^65^.

### DGE-RNAseq

The DGE RNA sequencing was performed following the published method^39,40^ with modifications as described below. MCF7 cells were seeded in a 384 well plate at 2500 cells per well, allowed to adhere for 24 hours, then treated with two doses in triplicate of XL177A, XL177B, Nutlin-3, or DMSO. After 24 hours of treatment, an EL405x plate washer (BioTek) was used to aspirate the media and wash once with PBS. Cells were lysed in the plate with 10 µl of lysis buffer (1x Qiagen TCL, 1% BME) for 5 minutes at room temperature, then stored at −80 °C. Automated liquid handling was performed with assistance from ICCB-Longwood Screening Facility. A BRAVO Automated Liquid Handling Platform (Agilent) was used for RNA extraction as follows. The lysate was mixed and 10 µl was transferred to a 384 well PCR plate. 28 µl of homemade SPRI^66^ (solid-phase reversible immobilization) beads were added to the lysate and mixed. After 5 minutes, the beads were pulled down by placing the plate on a magnet, then the beads were washed twice with 80% ethanol. Beads were air dried for 1 minute, then 20 µl of nuclease free water was added, the plate was removed from the magnet, and the beads were resuspended. After 5 minutes, the plate was placed back on the magnet to pull down the beads. The supernatant was transferred to a fresh 384 well plate. RNA quantity was checked with the Qubit Fluorometer and RNA quality was assessed using the Agilent BioAnalyzer RNA 6000 Pico Kit. The BRAVO platform was again used to transfer 5 µl of supernatant was transferred to a fresh 384 well plate with RT master mix and 1 µl of barcoded E3V6NEXT adapters for reverse transcription and template switching. The plate was incubated for 90 minutes at 42 °C, then cDNA was pooled, purified with the QIAquick PCR purification kit. The full-length cDNA was treated with Exonuclease I to remove excess primers for 30 minutes at 37 °C then amplified x5 cycles with Advantage 2 PCR Enzyme System using the SINGV6 primer. The amplified full-length cDNA was purified with Agencourt AMPure XP magnetic beads and quantitated by Qubit Fluorometer. The sequencing library was prepared using the Nextera DNA kit following the manufacturer’s instructions. Briefly, 55 ng of cDNA was tagmented for 5 minutes at 55 °C then purified using Zymo DNA Clean & Concetrator-5 column, then amplified x7 cycles and purified using a 0.9x ratio of AMPure XP magnetic beads. The library size distribution was assessed by Agilent BioAnalyzer HS DNA Kit before it was quantified by qPCR and sequenced on an Illumina NextSeq at the Biopolymers Facility at Harvard Medical School.

#### Analysis

The bcbio-nextgen single cell RNA-seq analysis pipeline (https://bcbio-nextgen.readthedocs.io/en/latest/) was used to deconvolve the well barcodes and convert reads to counts. Any detected barcode that differed by more than one base from an expected barcode was removed. Unique reads were identified using unique molecular identifiers (UMIs) in order to remove PCR duplicates. The reads remaining after these filters were aligned to the transcriptome (GRCh38) using RapMap^67^. The R package DESeq. 2 version 1.20.0 (R version 3.5.1) was used for differential expression analysis^68^. The Molecular Signatures Database (v6.2)^41,69,70^ was used to compute overlap for the significantly upregulated and downregulated genes (adjusted p value <0.05 and fold change> 1.5) for XL177A and Nutlin-3 at both doses.

### Cancer cell line profiling

Cancer cell line profiling was performed using the Broad’s PRISM platform. Cell treatment and data analysis were performed as described in Yu et al^42^. Raw Luminex signal was converted to AUC values for each cell line as described. The resulting AUC profile for each compound was modeled against DepMap using the limma package in R^71^. Because hotspot mutation analysis was performed against DepMap, *TP53* annotations in Fig. 6c,d are directly from DepMap. *TP53* annotation using the functional scores in Giacomelli et al^44^. also produced significant differences between the functional WT and functional mutant populations (XL177A: mean diff. = 0.072 ± 0.016, p < 0.0001; Nutlin-3: mean diff. = 0.165 ± 0.011, p < 0.0001).

### Cell viability

Cells were plated in 384-well culture-treated plates and allowed to settle overnight. After drug treament and appropriate incubation time, cell viability was assessed using the CellTiter-Glo Luminescent Cell Viability Assay (Promega). Luminescence was read on a Fluostar Omega Reader (BMG Labtech).

### Computational cancer profiling

The Broad Institute’s public DepMap portal was accessed via www.depmap.org/portal/interactive. The top 100 gene profile correlations for *USP7* CRISPR (Avana) and Combined RNAi (Broad, Novartis, Marcotte) were downloaded directly from the DepMap portal. *TP53* profiling was performed offline after downloading the CRISPR (Avana) and Combined RNAi (Broad, Novartis, Marcotte) gene dependency sets. *TP53* annotation was performed manually using the methods in Giacomelli *et al*.^44^, with cell lines with functional scores> 0 annotated as *TP53* functional WT and cell lines with functional scores <0 annotated as *TP53* functional mutant. Parametric, unpaired, two-tailed t-tests were performed using GraphPad PRISM.

### Dual reporter luciferase competition assay

p53^WT^ and p53^NULL^ A549 cells constitutively expressing firefly luciferase or Renilla luciferase have been described^44^. Each cell line was infected with lentivirus encoding *S. pyrogenes* Cas9 under control of the human EF1alpha promoter (pLX311) and selected in blasticidin (InvivoGen) (1 mg/mL) (10 μg/mL). To perform the competition assay, Cas9-expressing p53^WT^ cells were mixed at a 1:1 ratio with complementarily-labeled Cas9-expressing p53^NULL^ cells and seeded at 2,500 cells/well in 96-well dishes in 200 µL of normal culture media. The following day, cells were infected with an array of sgRNA-expressing lentiviruses (pXPR003). Twenty-four hours thereafter, the supernatant was removed and fresh media containing puromycin (InvivoGen) (1 μg/mL) was added to select for infected cells. Two days later, cells were split into two new replica plates, and incubated for four more days. One replica plate was subjected to a dual luciferase assay^72^ and luminescence readings were obtained using a Wallac EnVision (Perkin-Elmer). Readings from wells infected with experimental sgRNAs were normalized to wells infected with control sgRNAs, and firefly:Renilla luminescence ratios were calculated to estimate the relative effects of sgRNAs on p53^WT^ versus p53^NULL^ cells within a well. To continue the assay, the second replica plate was passaged at a 1/16 dilution^44^. The process of reading and re-plating the cells was repeated every 4 days.

### Chemistry

All commercially available starting materials were purchased from *Sigma Aldrich*, *Fisher Scientific*, *Oakwood Chemical* and *Combi Blocks*, and anhydrous solvents were purchased from *Fisher Scientific*. All reagents and solvents were used as received without further purification. If necessary, air or moisture sensitive reactions were carried out under an inert nitrogen atomsphere.

Removal of solvents was accomplished on a Büchi R-300 rotary evaporator connected to a *Welch* 1400B-01 vacuum line and *Labconco FreeZone 6* plus system. Compound purification was achived using normal phase column chromatography (Teledyne CombiFlash chromatography system) and/or reversed phase chromatography (Shimadzu system with SunFire^®^ Prep C_18_ OBD^TM^ 5 μM column). Purity was assessed by UPLC (Waters Acquity system) and analytical thin layer chromatography (TLC, EMD Millipore TLC Silica Gel60 F254). TLC visualization was accomplished by irradiation under UV light (254 nm).

All ^1^H-NMR spectra were recorded at 298 K on a Bruker ARX 500 (500 MHz) spectrometer, and all ^13^C-NMR spectra were recorded on a Bruker ARX 500 (126 MHz) spectrometer. Samples were dissolved in CDCl_3,_ DMSO-*d*6, or CD_3_OD, and spectra were referenced to the residual solvent peak (chlorofrom-*d*: 7.26 ppm for ^1^H-NMR and 77.16 ppm for ^13^C-NMR; DMSO-*d*6: 2.50 ppm for ^1^H-NMR and 39.25 ppm for ^13^C-NMR, CD_3_OD: 3.31 ppm (-Me) for ^1^H NMR and 49.00 ppm for ^13^C NMR or tetramethylsilane (TMS) as the internal standard. NMR peaks are reported below with chemical shift, multiplicity (s = singlet, d = doublet, t = triplet, q = quartet, m = multiplet, br = broad peak), coupling constants (Hz), and number of protons. Mass spectrometry (LCMS) data were obtained on Waters Acquity UPLC system in positive ESI mode.

For synthetic routes and analysis see Supplementary Methods and Figs. S15 and S16.

## Supplementary information

Supplementary information

Supplementary information2

Supplementary information3

Supplementary information4

Supplementary information5

Supplementary information6

## Data Availability

All data generated during this study are included in this article (and its Supplementary Files) or are available from the corresponding author upon request. A Life Sciences Reporting Summary for this paper is available.
