# Supplementary material for: Selective USP7 inhibition elicits cancer cell killing through a p53-dependent mechanism
Source: Sci Rep. 2020 Mar 24;10:5324. doi: 10.1038/s41598-020-62076-x (PMC7093416; doi:10.1038/s41598-020-62076-x)
Supplement: Supplementary file 1 — Supplementary information [file 41598_2020_62076_MOESM1_ESM.pdf]

Selective USP7 inhibition elicits cancer cell killing through a p53-dependent mechanism

Nathan J. Schauer[1,2,†], Xiaoxi Liu[1,2,†], Robert S. Magin[1,2], Laura M. Doherty[1,2,3], Wai Cheung Chan[1,2], Scott B. Ficarro[1,13], Wanyi Hu[1], Rebekka M. Roberts[1], Roxana E. Iacob[4], Björn Stolte[5,6,7], Andrew O. Giacomelli[7,8,9], Sumner Perera[10], Kyle McKay[11], Sarah A. Boswell[3], Ellen L. Weisberg[8], Arghya Ray[8,12], Dharminder Chauhan[8,12], Sirano dhe Paganon[1], Ken C. Anderson[8,12], James D. Griffin[8], Jianing Li[11], William C. Hahn[7,8,9], Peter K. Sorger[3], John R. Engen[4], Kimberly Stegmaier[5,7], Jarrod A. Marto[1,13], Sara J. Buhrlage[1,2,*]

[1] Department of Cancer Biology and the Linde Program in Cancer Chemical Biology, Dana-Farber Cancer Institute, Boston, MA, USA
[2] Department of Biological Chemistry and Molecular Pharmacology, Harvard Medical School, Boston, MA, USA
[3] Department of Systems Biology and Laboratory of Systems Pharmacology, Harvard Medical School, Boston, MA, USA
[4] Department of Chemistry and Chemical Biology, Northeastern University, Boston, MA, USA
[5] Department of Pediatric Oncology, Dana-Farber Cancer Institute and Boston Children's Hospital, Boston, MA, USA
[6] Dr. von Hauner Children's Hospital, Department of Pediatrics, University Hospital, LMU Munich, Munich, Germany
[7] The Broad Institute of MIT and Harvard University, Cambridge, MA, USA
[8] Department of Medical Oncology, Dana-Farber Cancer Institute, Boston, MA, USA
[9] Department of Medicine, Harvard Medical School, Boston, MA, USA
[10] Harvard College, Cambridge, MA, USA
[11] Department of Chemistry, University of Vermont, Burlington, VT, USA
[12] The LeBow Institute for Myeloma Therapeutics and Jerome Lipper Myeloma Center, Dana-Farber Cancer Institute, Boston, MA, USA
[13] Department of Oncologic Pathology and Blais Proteomics Center, Dana-Farber Cancer Institute, Boston, MA, USA
[†] These authors contributed equally to this work
* e-mail: saraj_buhrlage@dfci.harvard.edu

Supplementary Data

Supplementary Figures S1 – S16

Supplementary Synthetic Methods

Supplementary Figure Captions

Figure S1: Full-length recombinant purified USP7 was pre-incubated with XL041 at the indicated concentrations for the indicated times, then Ub-AMC was added and fluorescence was monitored. Graphpad Prism software was used to calculate initial reaction velocity and derive K_I / k_{inact} values (bottom panel).

Figure S2: Mass spectra (left) and zero-charge mass spectra (right) of WT USP7 catalytic domain incubated for 4 hours with DMSO (top) or 2.5-fold molar excess XL058 (bottom).

Figure S3: Mass spectra (left) and zero-charge mass spectra (right) of USP7 C223A catalytic domain incubated for 2 hours with DMSO (top) or 2.5-fold molar excess XL177A (bottom).

Figure S4: Representative flow cytometry gating for cell cycle analysis of MCF7 cells treated with DMSO, serum starvation, 1 μM taxol, 1 μM XL177A, or 1 μM XL177B for 24 hours. Each plot is one of three experimental replicates for each condition.

Figure S5: MCF7 cells were transfected with the indicated pcDNA constructs for 48 hours, then treated with XL177A at the indicated concentration for 3 hours. Cells were lysed, and FLAG-USP7 target engagement was assessed with the DUB ABP HA-Ub-VS. Densitometry was performed using ImageStudio software and analyzed using Graphpad Prism.

Figure S6: HDX data. **A.** USP7 coverage map showing the common peptic peptides that were compared between free USP7 and XL041 USP7 bound, and the deuterium incorporation plots for all 81 peptic peptides that were compared between USP7 free (red) and XL041bound (blue). On the x axis is the time in minutes and on the y-axis is the relative uptake (Da). The maximum y-axis value for each plot represents the theoretical maximum amount of D that can be incorporated into a peptide. Values represent the mean of two individual measurements; error bars, s.d. **B**. USP7 coverage map showing all the common peptic peptides that were compared between free USP7 and XL177A USP7 bound, and the deuterium incorporation plots for all 105 peptic peptides that were compared between USP7 free (red) and XL177A bound (blue). **C.** Chiclet representation of the HDX MS data. The peptides from panel A and B are represented from N to C terminus (top to bottom) at each time point (left to right). The deuterium level for each bound state was subtracted from

that of the protein alone and colored according to the legend shown. The horizonal dotted lines are placed to help orient the readers to notice the regions of difference between the two states. Note that in the HDX MS data set the protein sequence numbering is different and Cys34 is actually Cys223.

Figure S7. Snapshot of the XL188-USP7 construct (red) and the XL177A-USP7 construct (orange) superimposed on each other after 150 ns of simulation time shows the benzyl moiety of XL177A in a similar confirmation to that of XL188.

Figure S8: DUBs identified by ABPP on HEK293 lysate when probed with 1 μM of the indicated probe, with a 1% FDR cut-off.

Figure S9: Heatmaps showing correlation between XL177A and XL177B overall viability among all cell lines screened in PRISM at the indicated doses. XL177A = K87132179; XL177B = K79243224

Figure S10: PRISM results from XL177B (top) and Nutlin-3A (bottom) showing correlations with CRISPR profiling (left), mutational profiling (center), and lineage enrichment (right).

Figure S11: Cellular proliferation of panels of acute myeloid leukemia (AML); soft tissue sarcomas including two rhabdoid (G401, G402) and one uterine sarcoma (MES-SA); and multiple myeloma cell lines treated with XL177A (left) or XL177B (right). Red data points indicated *TP53*-WT cell lines, and black data points indicate *TP53* mutants.

Figure S12: Top Panel: The indicated Ewing Sarcoma cell lines were treated with XL177B or GNE-6640 for 3 days, and viability was assessed by Cell Titer Glo. Red lines indicate p53-WT cells, and black lines indicate p53-mutant cells. Bottom Panel: TC32 cells stably expressing the indicated sgRNA was treated for 3 days with XL177B or GNE-6640 at the indicated doses, and viability was assessed by Cell Titer Glo.

Figure S13: A 1:1 mixture of A549 (top panel) or RKO (bottom panel) cells expressing FF and Renilla-sgTP53 were treated with sgRNAs targeting the indicated genes, then allowed to grow for 17 days, with luminescence readings taken at the indicated timepoints. WT:KO ratio is equivalent to the FF:Renilla ratio.

Figure S14: Original Western blots of figures in main text.

Figure S15: The general synthetic route used to synthesize XL177A, XL177B, XL112, XL041, and XL058.

Figure S16: The synthetic route used to synthesize XL024.

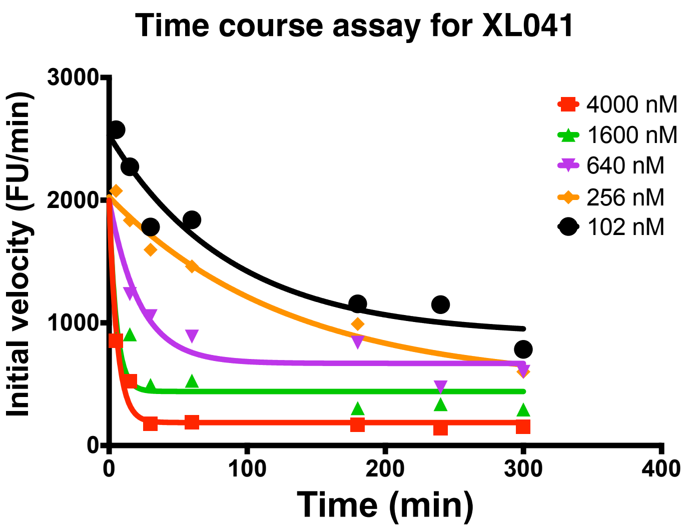

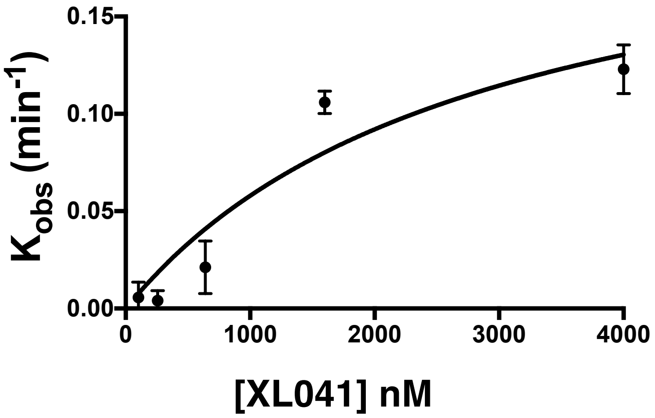

**Kinact (min-1)**	0.2237±0.07434
**Ki (μM)**	2.8±1.8

Figure S1

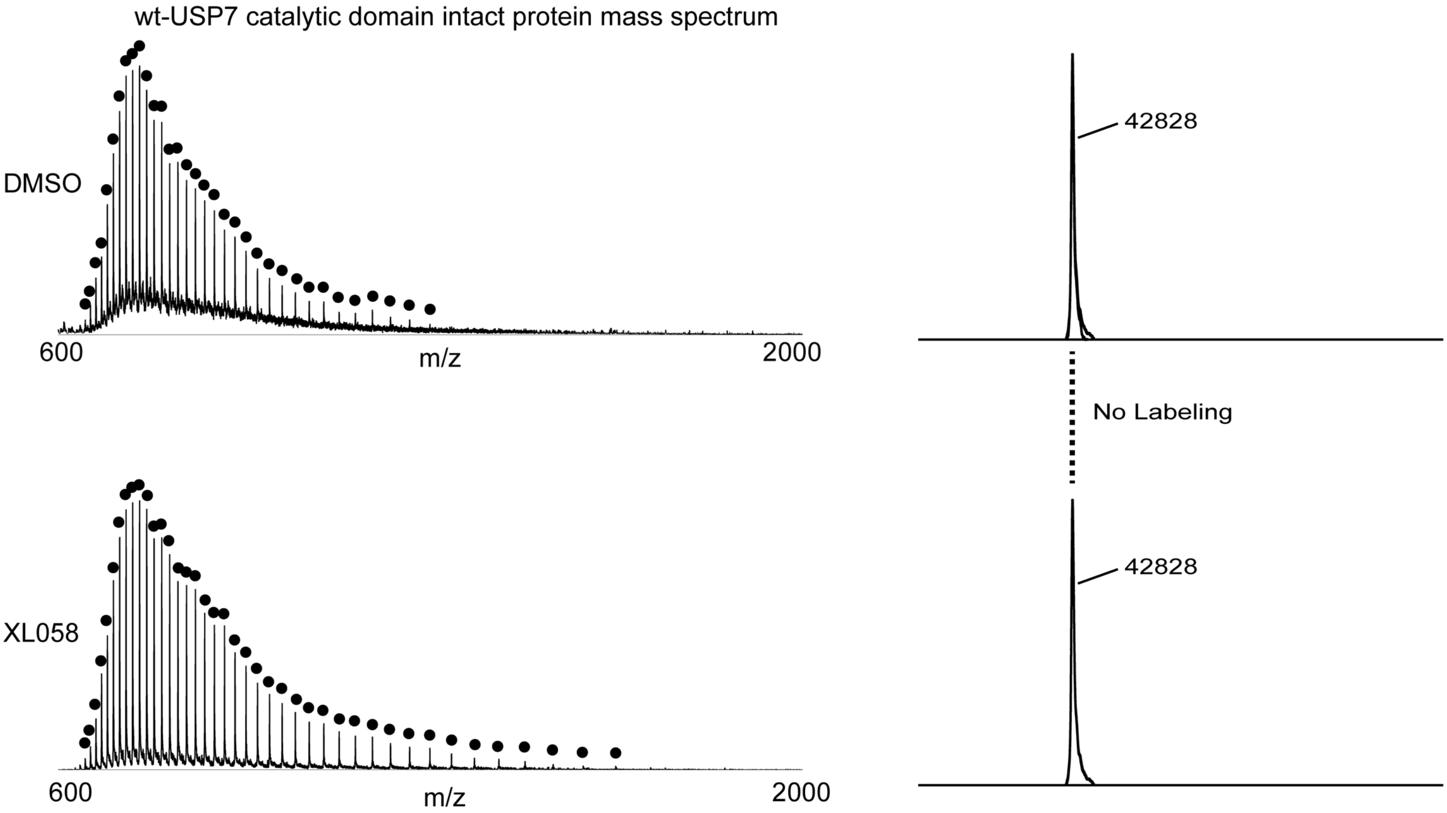
wt-USP7 catalytic domain intact protein mass spectrum
DMSO
600
m/z
2000
42828
No Labeling
XL058
600
m/z
2000
42828

Figure S2

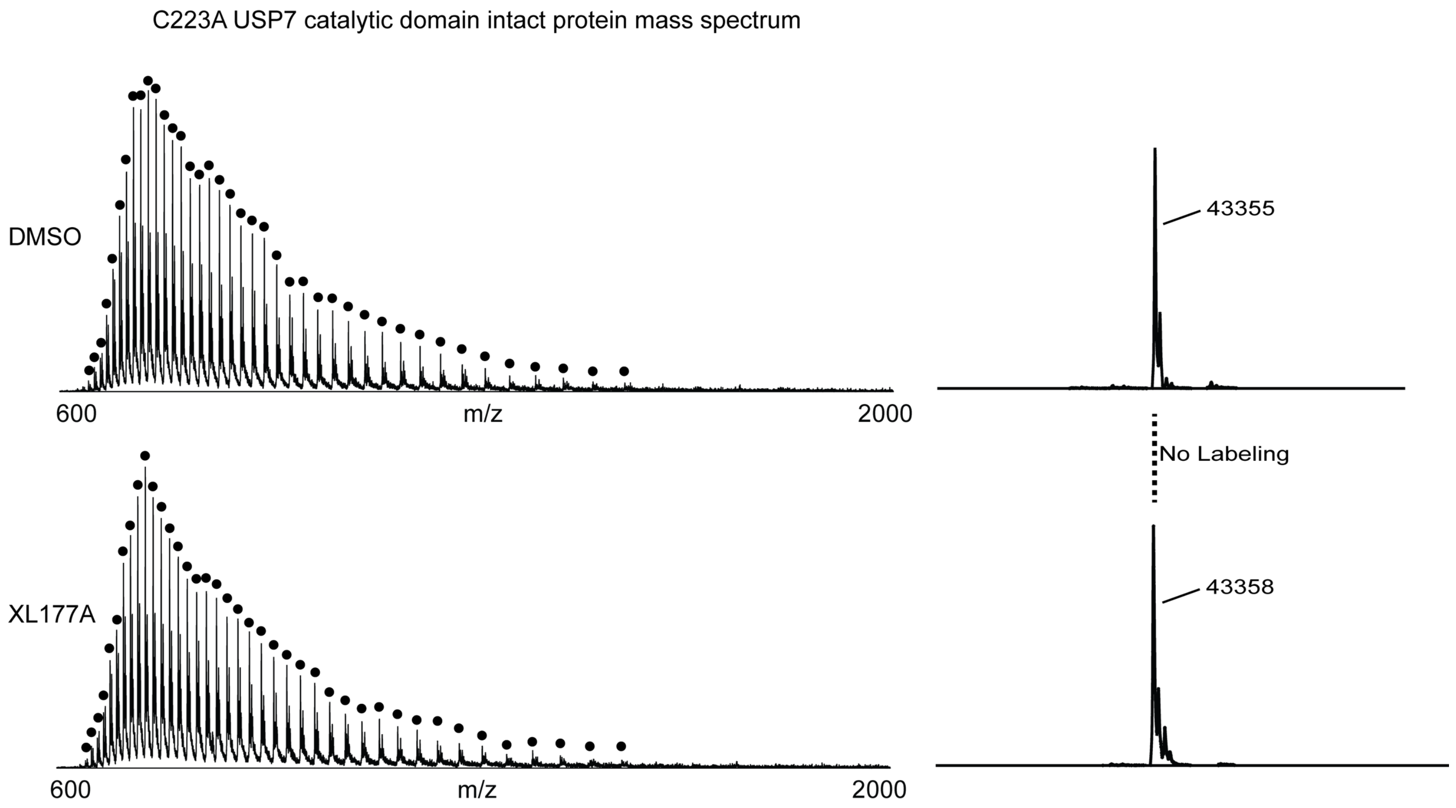
C223A USP7 catalytic domain intact protein mass spectrum
DMSO
600
m/z
2000
43355
No Labeling
XL177A
600
m/z
2000
43358

Figure S3

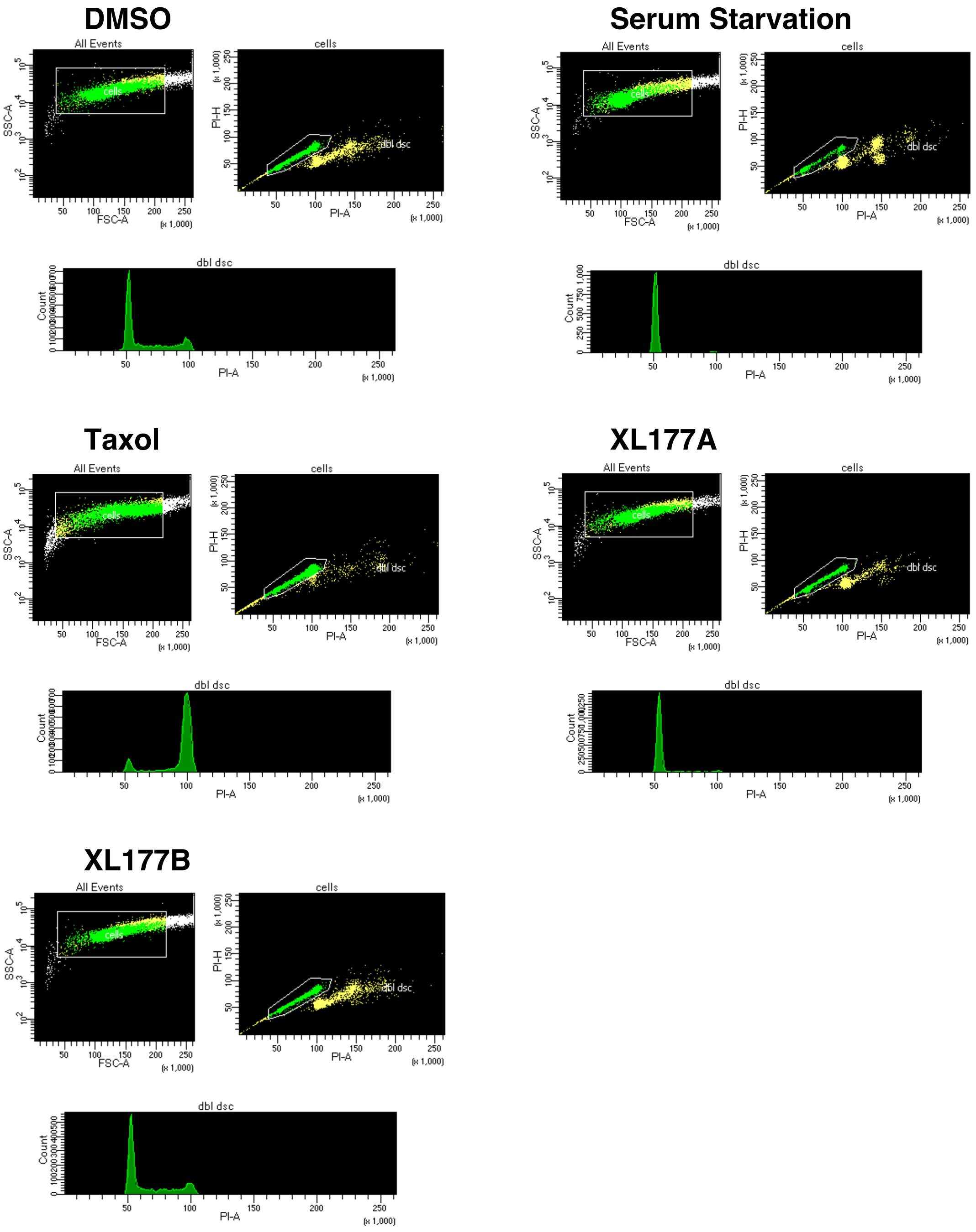
DMSO
Serum Starvation
Taxol
XL177A
XL177B
All Events
cells
dbl dsc
SSC-A
FSC-A
PI-H
PI-A
Count
(x 1,000)

Figure S4

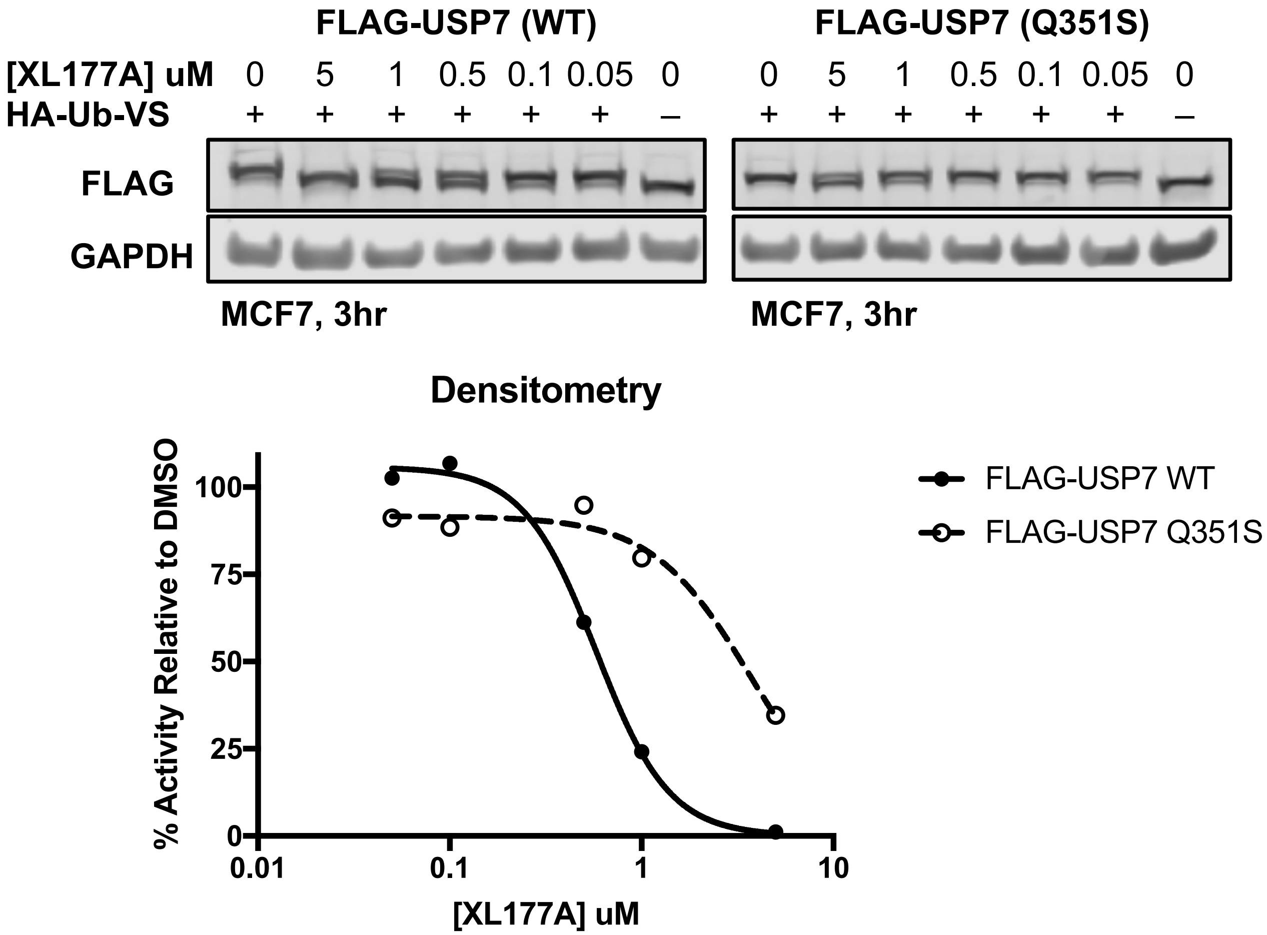
FLAG-USP7 (WT)
FLAG-USP7 (Q351S)
[XL177A] uM 0 5 1 0.5 0.1 0.05 0
HA-Ub-VS + + + + + + –
FLAG
GAPDH
MCF7, 3hr
MCF7, 3hr
Densitometry
% Activity Relative to DMSO
100
75
50
25
0
0.01
0.1
1
10
[XL177A] uM
FLAG-USP7 WT
FLAG-USP7 Q351S

Figure S5

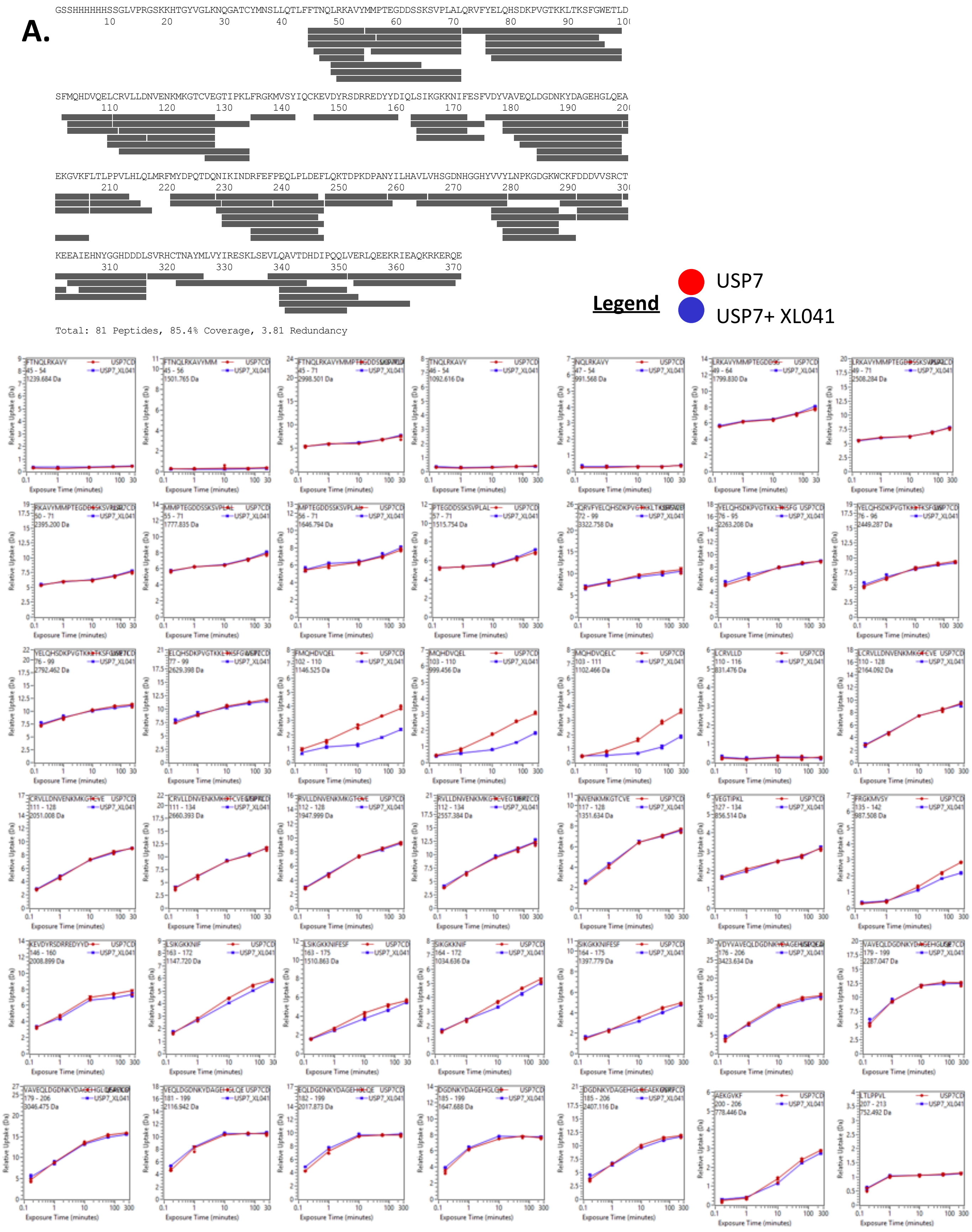
A.
GSSHHHHHHSSGLVPRGSKKHTGYVGLKNQGATCYMNSLLQTLFFTNQLRKAVYMMPTEGDDSSKSVPLALQRVFYELQHSDKPVGTKKLTKSFGWETLD
SFMQHDVQELCRVLLDNVENKMKGTCVEGTIPKLFRGKMVSYIQCKEVDYRSDRREDYYDIQLSIKGKKNIFESFVDYVAVEQLDGDNKYDAGEHGLQEA
EKGVKFLTLPPVLHLQLMRFMYDPQTDQNIKINDRFEFPEQLPLDEFLQKTDPKDPANYILHAVLVHSGDNHGGHYVVYLNPKGDGKWCKFDDDVVSRCT
KEEAIEHNYGGHDDDLSVRHCTNAYMLVYIRESKLSEVLQAVTDHDIPQQLVERLQEEKRIEAQKRKERQE
Total: 81 Peptides, 85.4% Coverage, 3.81 Redundancy
Legend
USP7
USP7+ XL041
Relative Uptake (Da)
Exposure Time (minutes)

A. continued

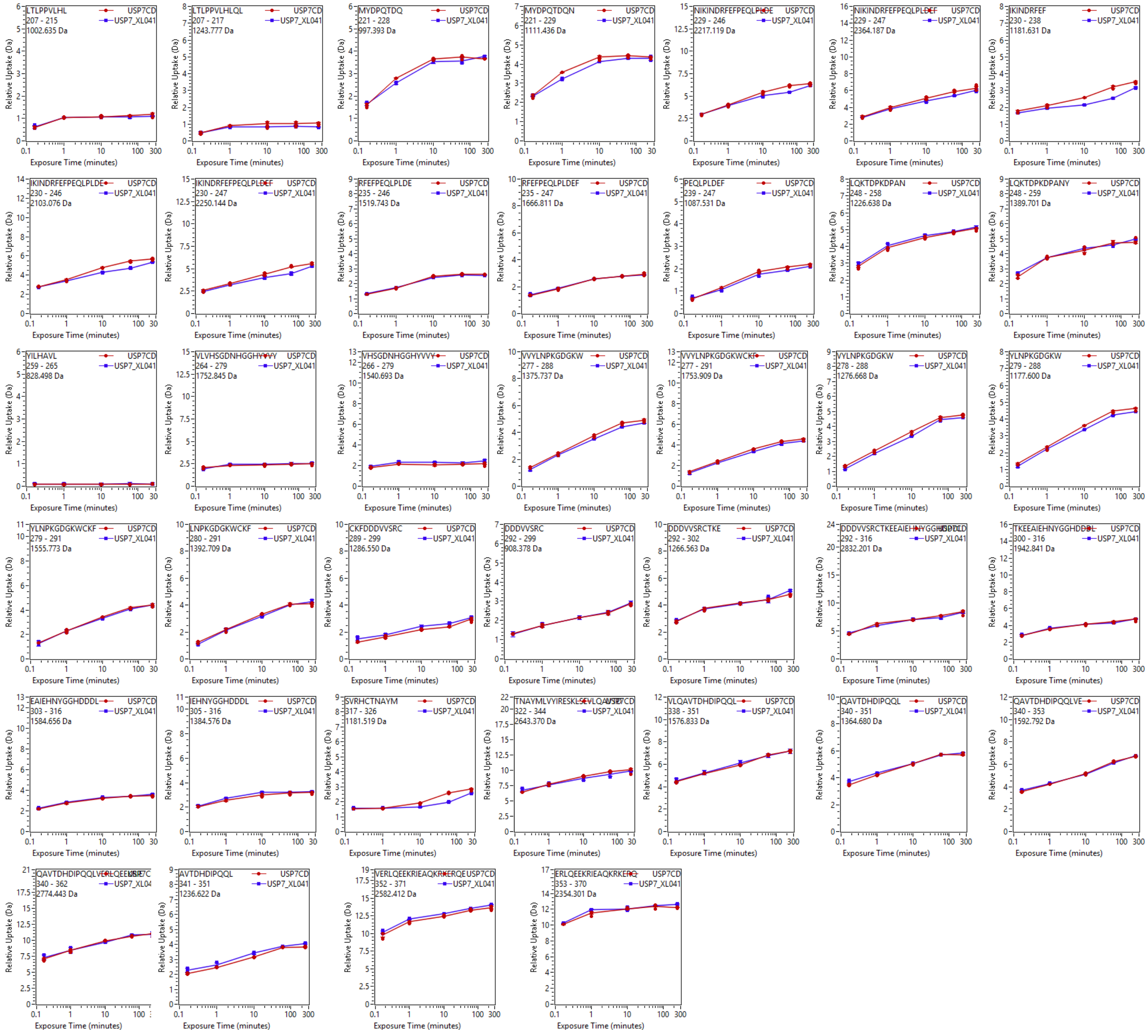
USP7CD
USP7_XL041
Relative Uptake (Da)
Exposure Time (minutes)
LTLPPVLHL 207 - 215 1002.635 Da
LTLPPVLHLQL 207 - 217 1243.777 Da
MYDPQTDQ 221 - 228 997.393 Da
MYDPQTDQN 221 - 229 1111.436 Da
NIKINDRFEFPEQLPLDE 229 - 246 2217.119 Da
NIKINDRFEFPEQLPLDEF 229 - 247 2364.187 Da
IKINDRFEF 230 - 238 1181.631 Da
IKINDRFEFPEQLPLDE 230 - 246 2103.076 Da
IKINDRFEFPEQLPLDEF 230 - 247 2250.144 Da
RFEFPEQLPLDE 235 - 246 1519.743 Da
RFEFPEQLPLDEF 235 - 247 1666.811 Da
PEQLPLDEF 239 - 247 1087.531 Da
LQKTDPKDPAN 248 - 258 1226.638 Da
LQKTDPKDPANY 248 - 259 1389.701 Da
YILHAVL 259 - 265 828.498 Da
VLVHSGDNHGGHYVVY 264 - 279 1752.845 Da
VHSGDNHGGHYVVY 266 - 279 1540.693 Da
VVYLNPKGDGKW 277 - 288 1375.737 Da
VVYLNPKGDGKWCKF 277 - 291 1753.909 Da
VYLNPKGDGKW 278 - 288 1276.668 Da
YLNPKGDGKW 279 - 288 1177.600 Da
YLNPKGDGKWCKF 279 - 291 1555.773 Da
LNPKGDGKWCKF 280 - 291 1392.709 Da
CKFDDDVVSRC 289 - 299 1286.550 Da
DDDVVSRC 292 - 299 908.378 Da
DDDVVSRCTKE 292 - 302 1266.563 Da
DDDVVSRCTKEEAIEHNYGGHDDDL 292 - 316 2832.201 Da
TKEEAIEHNYGGHDDDL 300 - 316 1942.841 Da
EAIEHNYGGHDDDL 303 - 316 1584.656 Da
IEHNYGGHDDDL 305 - 316 1384.576 Da
SVRHCTNAYM 317 - 326 1181.519 Da
TNAYMLVYIRESKLSEVLQAVTD 322 - 344 2643.370 Da
VLQAVTDHDIPQQL 338 - 351 1576.833 Da
QAVTDHDIPQQL 340 - 351 1364.680 Da
QAVTDHDIPQQLVE 340 - 353 1592.792 Da
QAVTDHDIPQQLVERLQEEK 340 - 362 2774.443 Da
AVTDHDIPQQL 341 - 351 1236.622 Da
VERLQEEKRIEAQKRKERQE 352 - 371 2582.412 Da
ERLQEEKRIEAQKRKERQ 353 - 370 2354.301 Da

B.

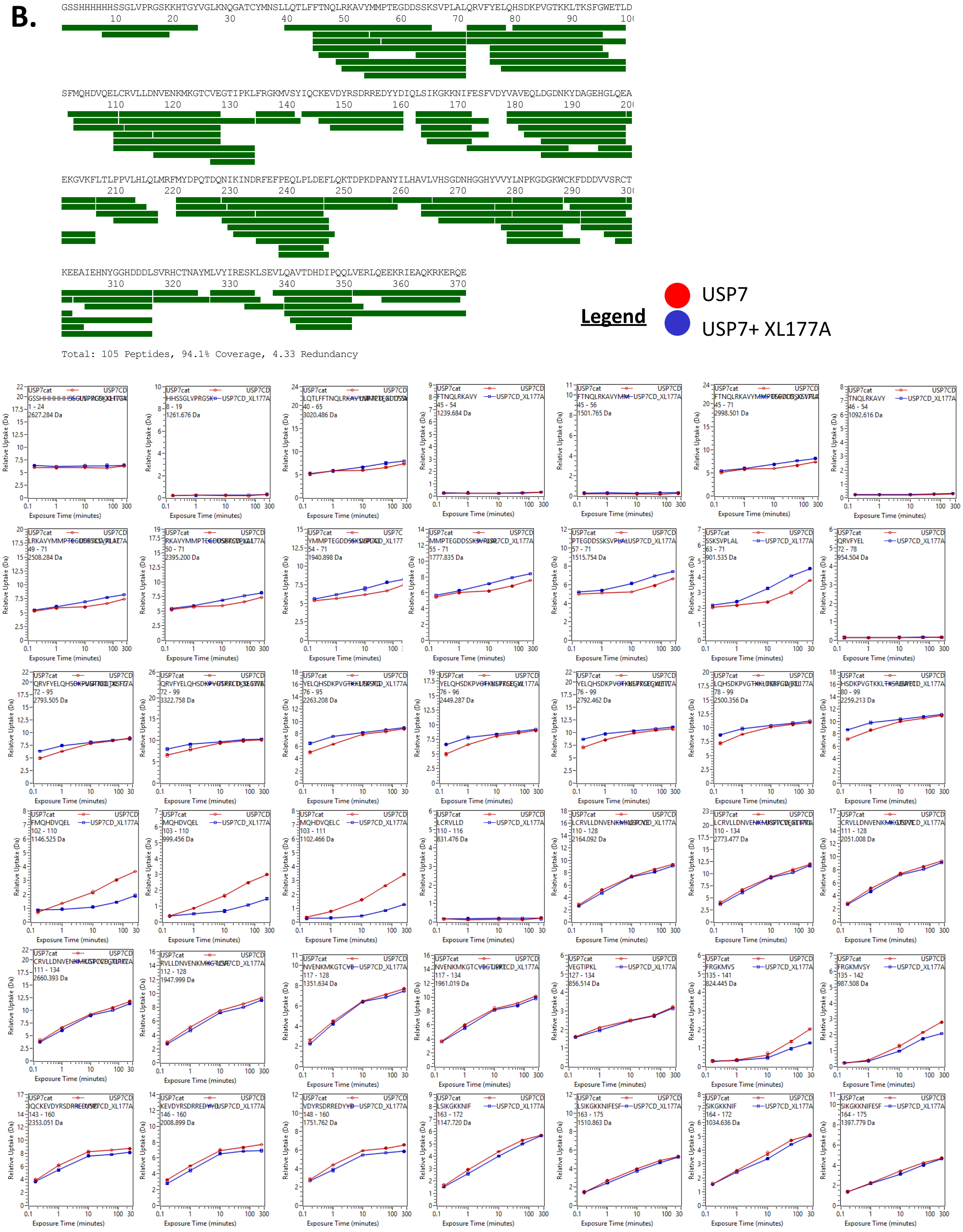
GSSHHHHHHSSGLVPRGSKKHTGYVGLKNQGATCYMNSLLQTLFFTNQLRKAVYMMPTEGDDSSKSVPLALQRVFYELQHSDKPVGTKKLTKSFGWETLD
SFMQHDVQELCRVLLDNVENKMKGTCVEGTIPKLFRGKMVSYIQCKEVDYRSDRREDYYDIQLSIKGKKNIFESFVDYVAVEQLDGDNKYDAGEHGLQEA
EKGVKFLTLPPVLHLQLMRFMYDPQTDQNIKINDRFEFPEQLPLDEFLQKTDPKDPANYILHAVLVHSGDNHGGHYVVYLNPKGDGKWCKFDDDVVSRCT
KEEAIEHNYGGHDDDLSVRHCTNAYMLVYIRESKLSEVLQAVTDHDIPQQLVERLQEEKRIEAQKRKERQE
Total: 105 Peptides, 94.1% Coverage, 4.33 Redundancy
Legend
USP7
USP7+ XL177A
Relative Uptake (Da)
Exposure Time (minutes)

B. continued

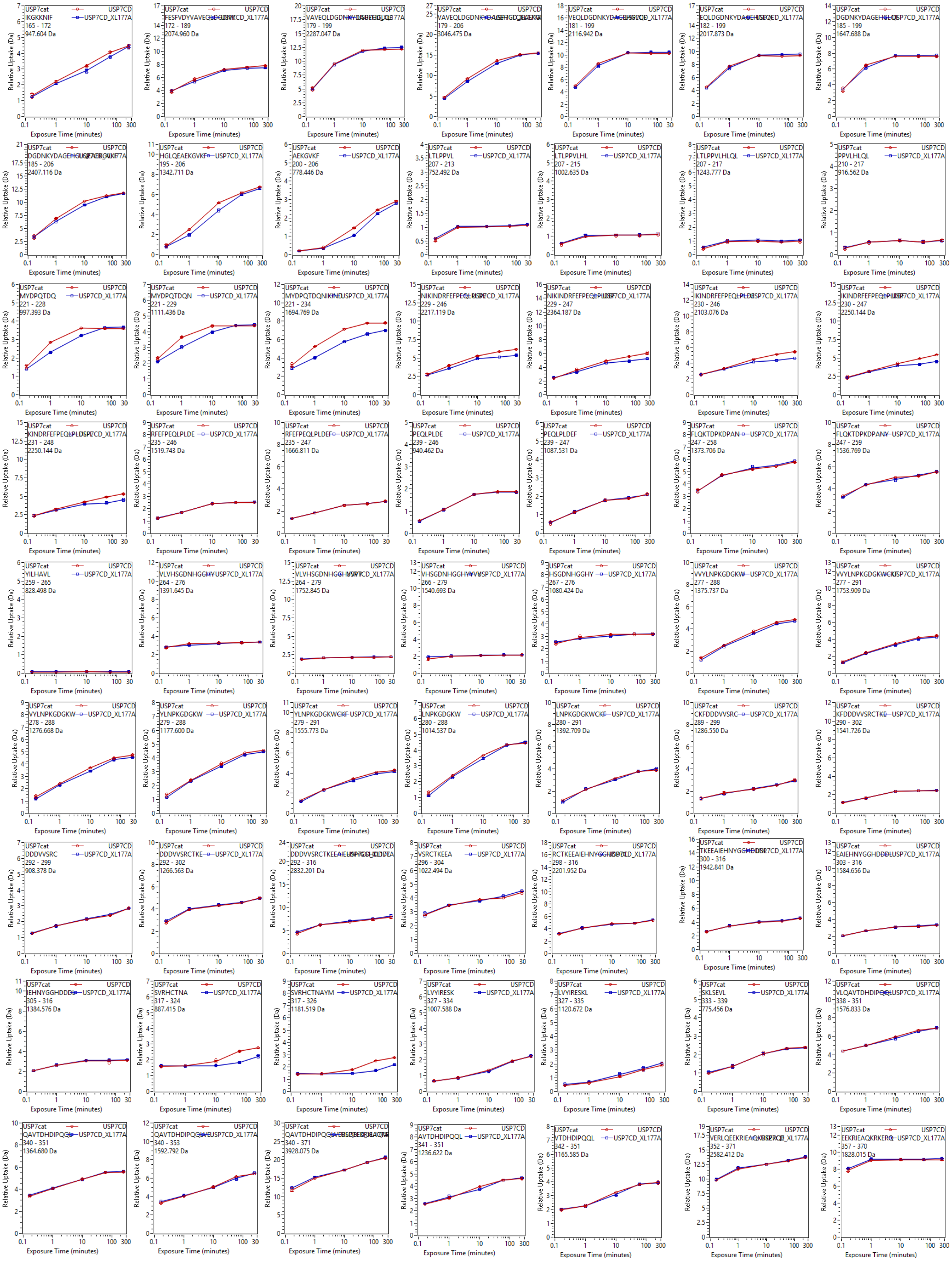
USP7cat
USP7CD_XL177A
Relative Uptake (Da)
Exposure Time (minutes)

C.

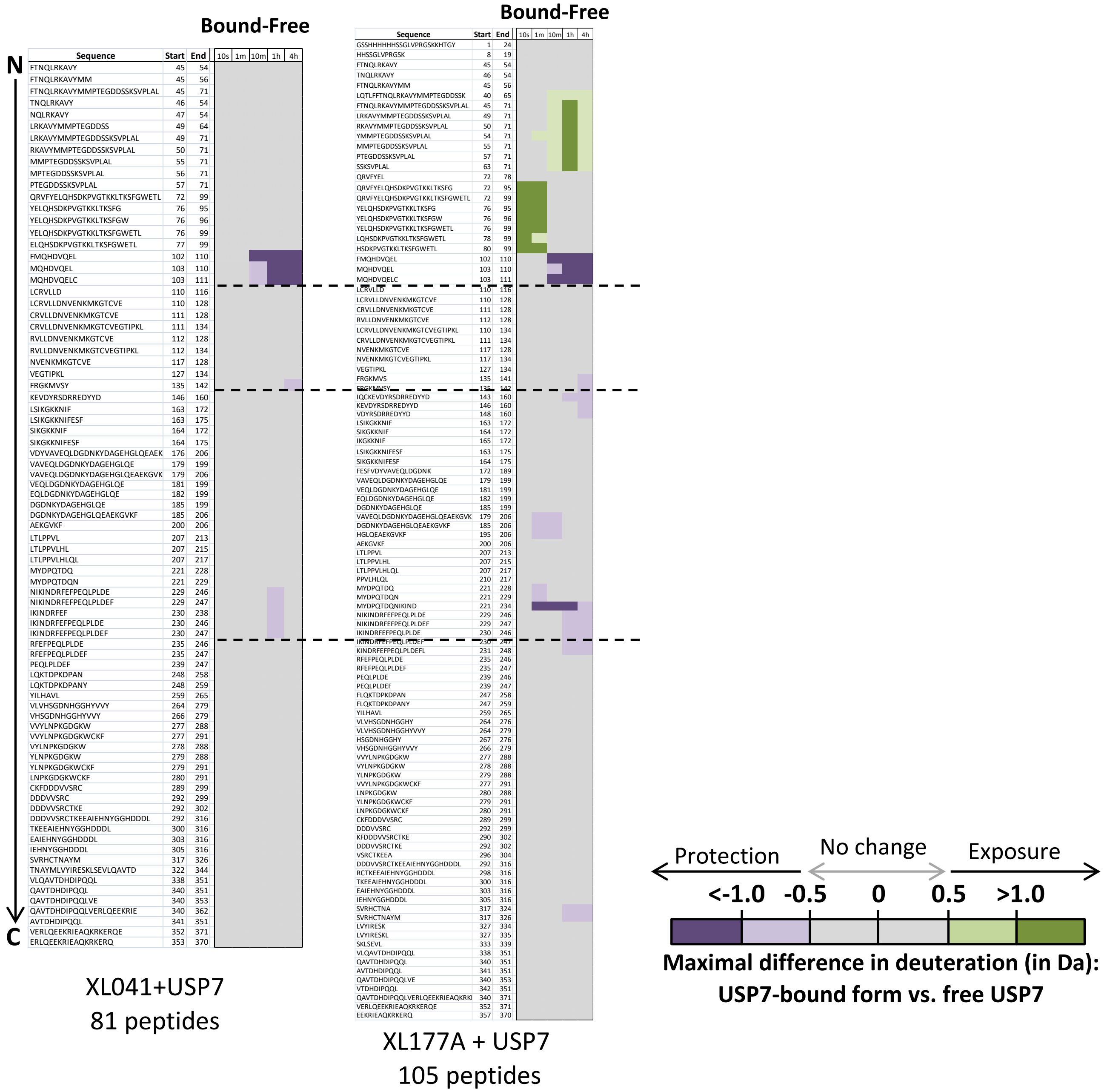
Bound-Free
Sequence
Start
End
10s
1m
10m
1h
4h
N
C
XL041+USP7
81 peptides
XL177A + USP7
105 peptides
Protection
No change
Exposure
<-1.0
-0.5
0
0.5
>1.0
Maximal difference in deuteration (in Da):
USP7-bound form vs. free USP7

Figure S6

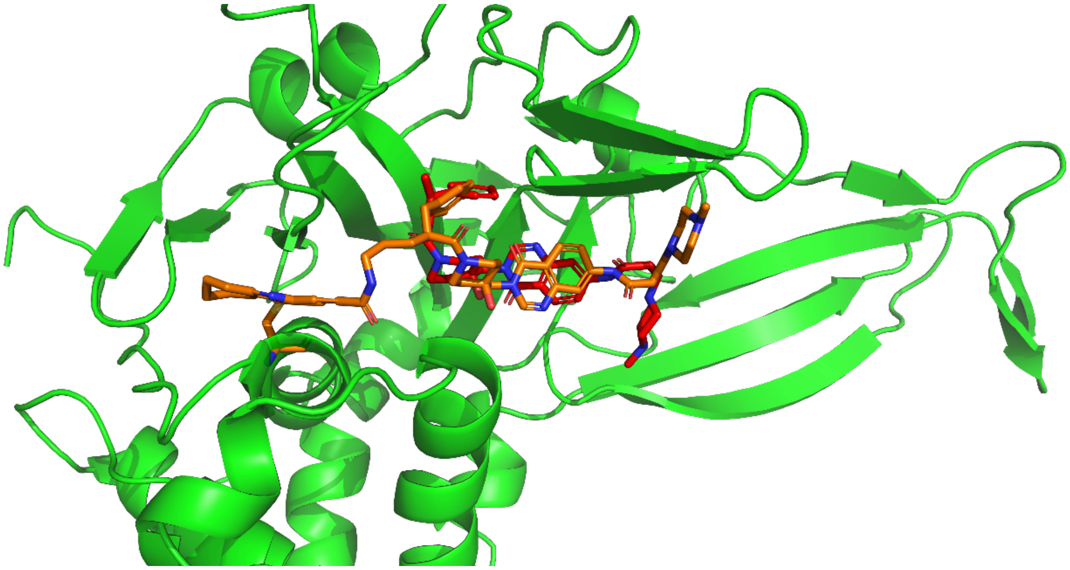

Figure S7

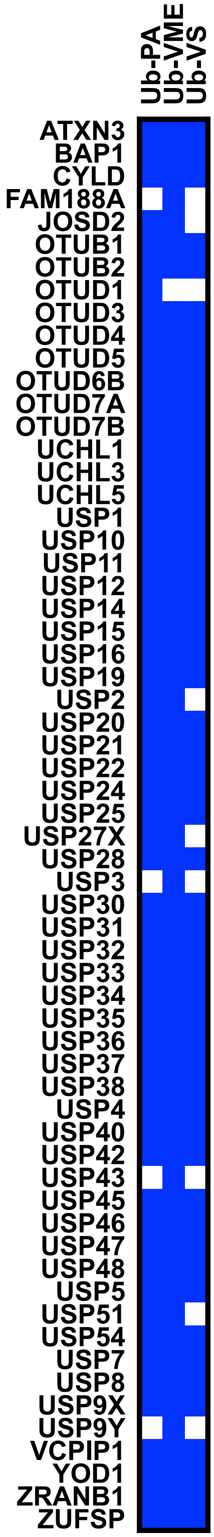
Ub-PA
Ub-VME
Ub-VS
ATXN3
BAP1
CYLD
FAM188A
JOSD2
OTUB1
OTUB2
OTUD1
OTUD3
OTUD4
OTUD5
OTUD6B
OTUD7A
OTUD7B
UCHL1
UCHL3
UCHL5
USP1
USP10
USP11
USP12
USP14
USP15
USP16
USP19
USP2
USP20
USP21
USP22
USP24
USP25
USP27X
USP28
USP3
USP30
USP31
USP32
USP33
USP34
USP35
USP36
USP37
USP38
USP4
USP40
USP42
USP43
USP45
USP46
USP47
USP48
USP5
USP51
USP54
USP7
USP8
USP9X
USP9Y
VCPIP1
YOD1
ZRANB1
ZUFSP

Figure S8

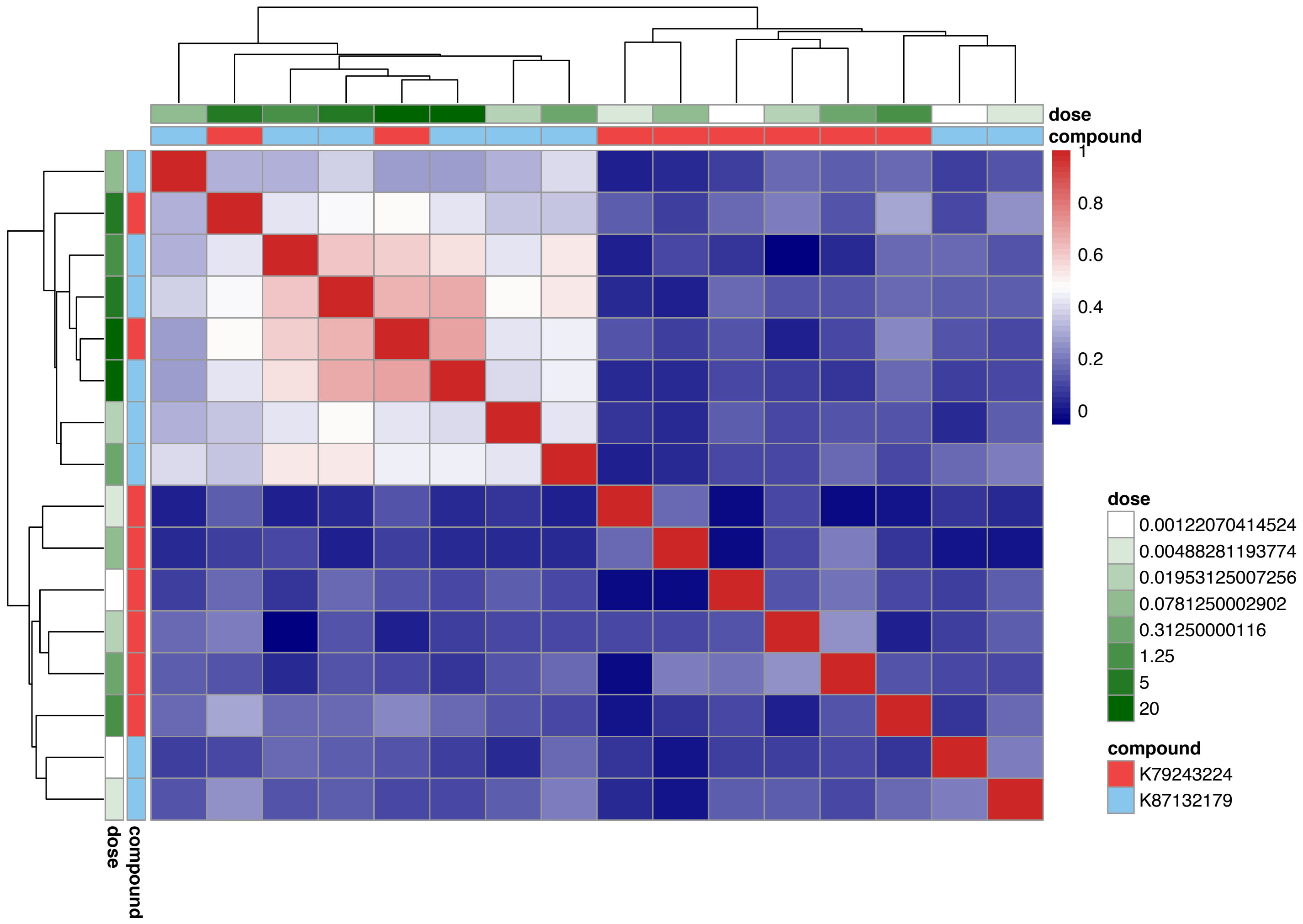
dose
compound
1
0.8
0.6
0.4
0.2
0
dose
0.00122070414524
0.00488281193774
0.01953125007256
0.0781250002902
0.31250000116
1.25
5
20
compound
K79243224
K87132179
dose
compound

Figure S9

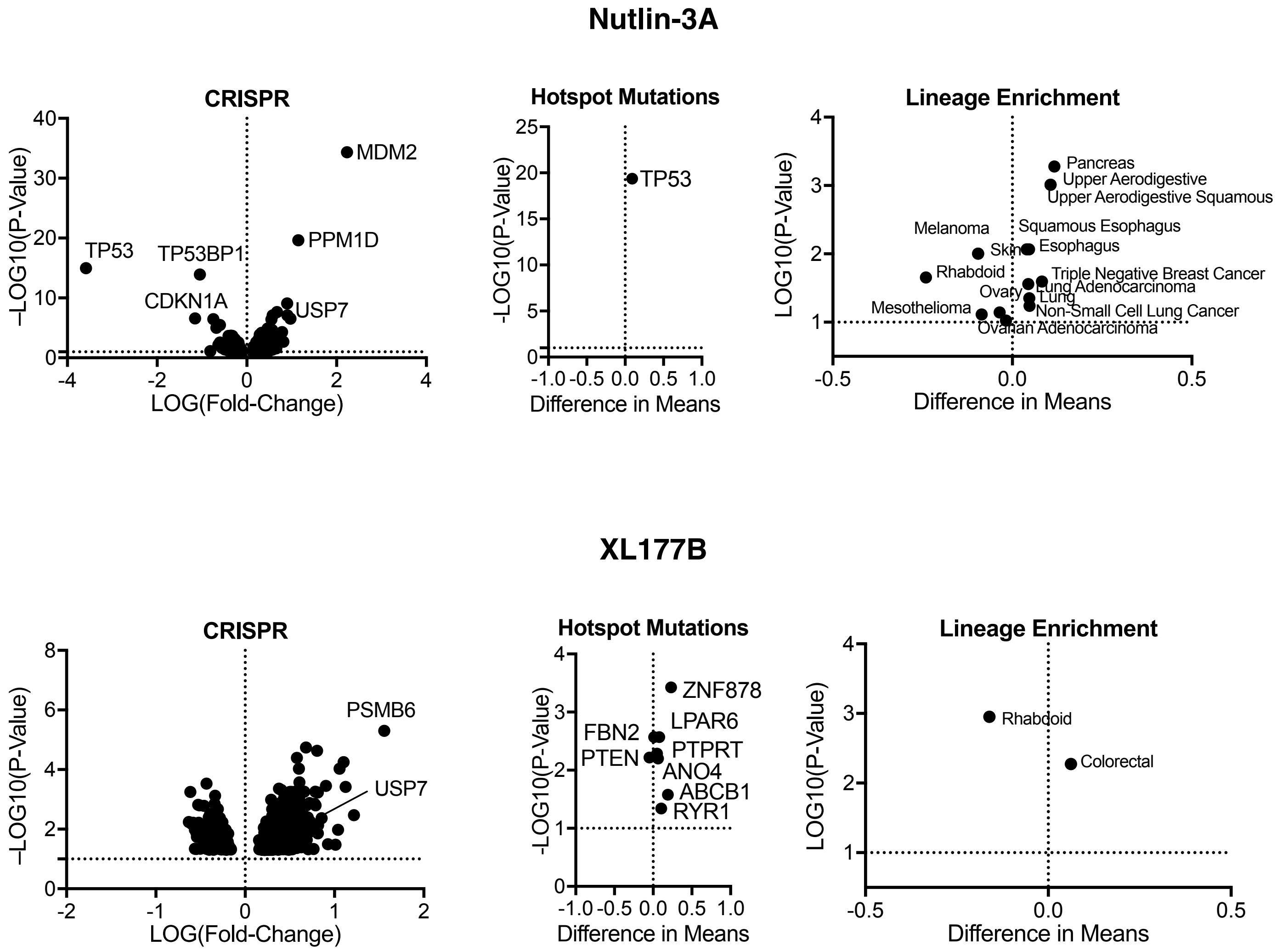

Nutlin-3A
CRISPR
Hotspot Mutations
Lineage Enrichment
−LOG10(P-Value)
LOG(Fold-Change)
MDM2
PPM1D
TP53
TP53BP1
CDKN1A
USP7
-LOG10(P-Value)
Difference in Means
TP53
LOG10(P-Value)
Pancreas
Upper Aerodigestive
Upper Aerodigestive Squamous
Melanoma
Squamous Esophagus
Skin
Esophagus
Rhabdoid
Triple Negative Breast Cancer
Ovary
Lung Adenocarcinoma
Lung
Mesothelioma
Non-Small Cell Lung Cancer
Ovarian Adenocarcinoma
XL177B
CRISPR
Hotspot Mutations
Lineage Enrichment
−LOG10(P-Value)
LOG(Fold-Change)
PSMB6
USP7
-LOG10(P-Value)
Difference in Means
ZNF878
LPAR6
FBN2
PTPRT
PTEN
ANO4
ABCB1
RYR1
LOG10(P-Value)
Rhabdoid
Colorectal

Figure S10

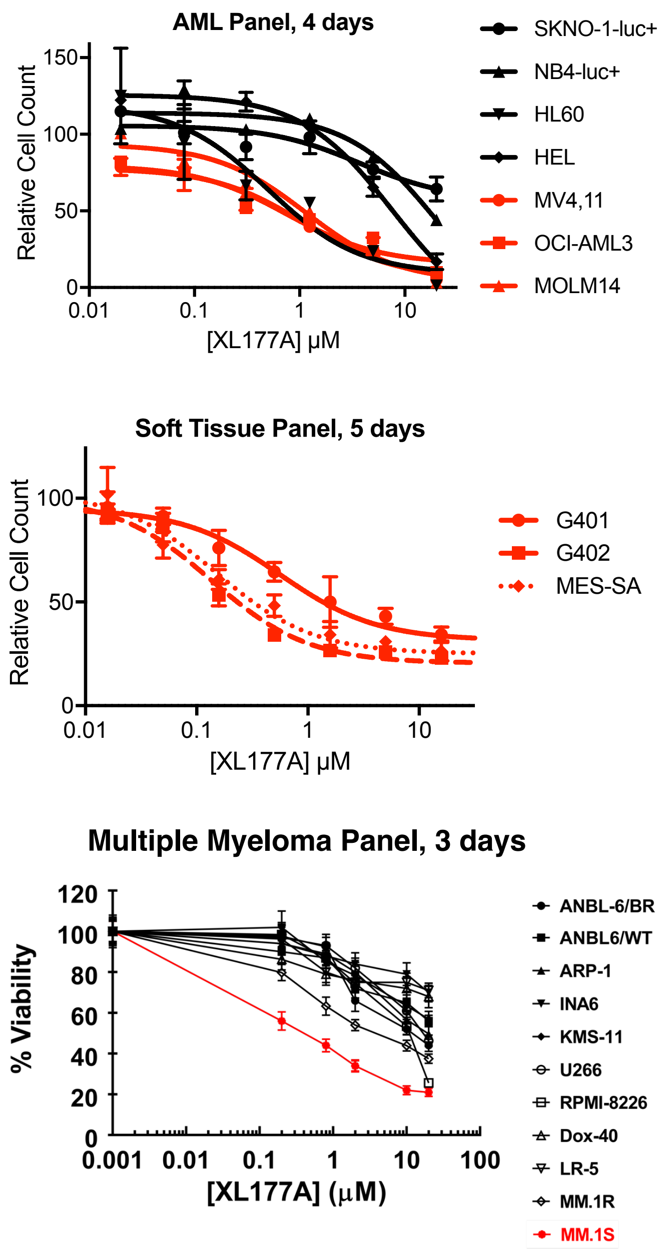

AML Panel, 4 days
Relative Cell Count
[XL177A] μM
SKNO-1-luc+
NB4-luc+
HL60
HEL
MV4,11
OCI-AML3
MOLM14
AML Panel, 4 days
Relative Cell Count
[XL177B] μM
Soft Tissue Panel, 5 days
Relative Cell Count
[XL177A] μM
G401
G402
MES-SA
Soft Tissue Panel, 5 days
Relative Cell Count
[XL177B] μM
Multiple Myeloma Panel, 3 days
% Viability
[XL177A] (μM)
ANBL-6/BR
ANBL6/WT
ARP-1
INA6
KMS-11
U266
RPMI-8226
Dox-40
LR-5
MM.1R
MM.1S

Figure S11

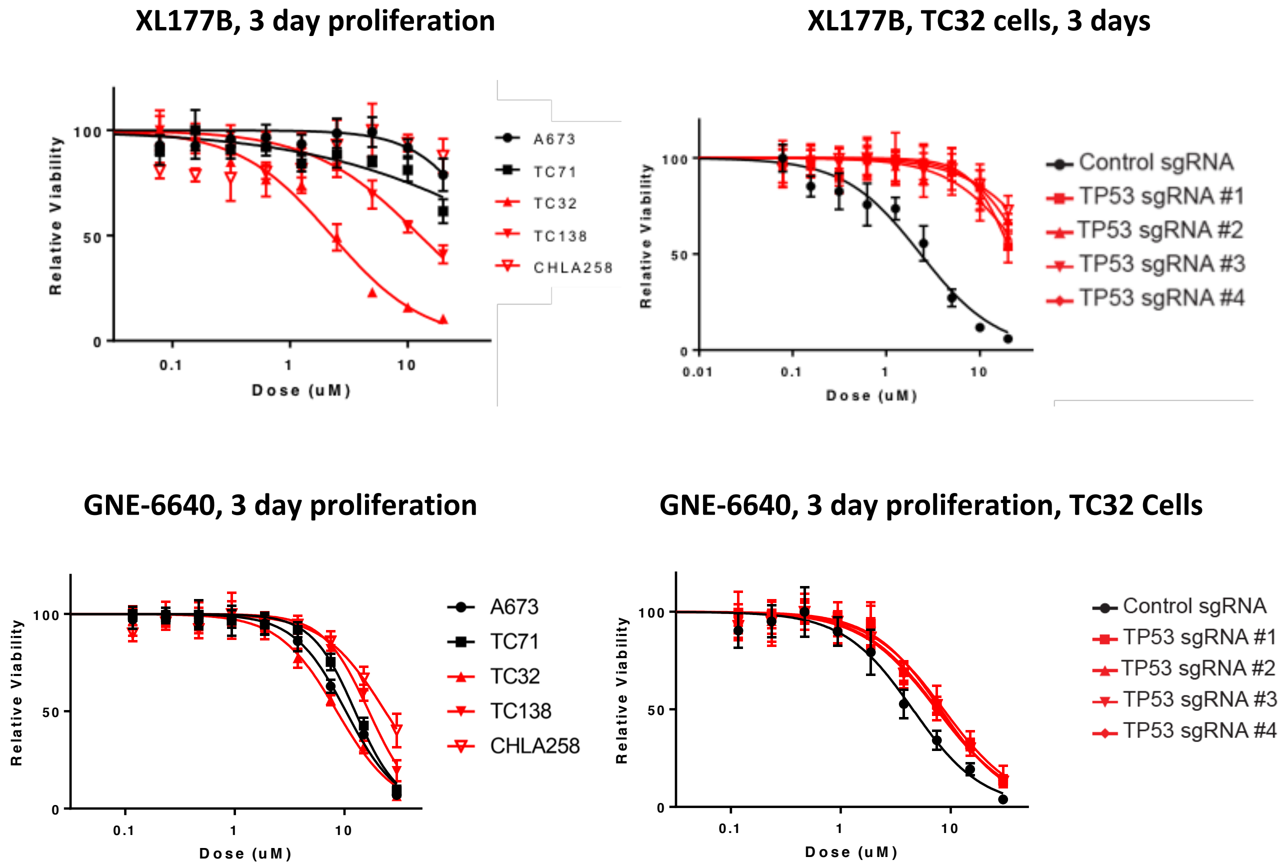
XL177B, 3 day proliferation
Relative Viability
Dose (uM)
A673
TC71
TC32
TC138
CHLA258
XL177B, TC32 cells, 3 days
Relative Viability
Dose (uM)
Control sgRNA
TP53 sgRNA #1
TP53 sgRNA #2
TP53 sgRNA #3
TP53 sgRNA #4
GNE-6640, 3 day proliferation
Relative Viability
Dose (uM)
A673
TC71
TC32
TC138
CHLA258
GNE-6640, 3 day proliferation, TC32 Cells
Relative Viability
Dose (uM)
Control sgRNA
TP53 sgRNA #1
TP53 sgRNA #2
TP53 sgRNA #3
TP53 sgRNA #4

Figure S12

A549

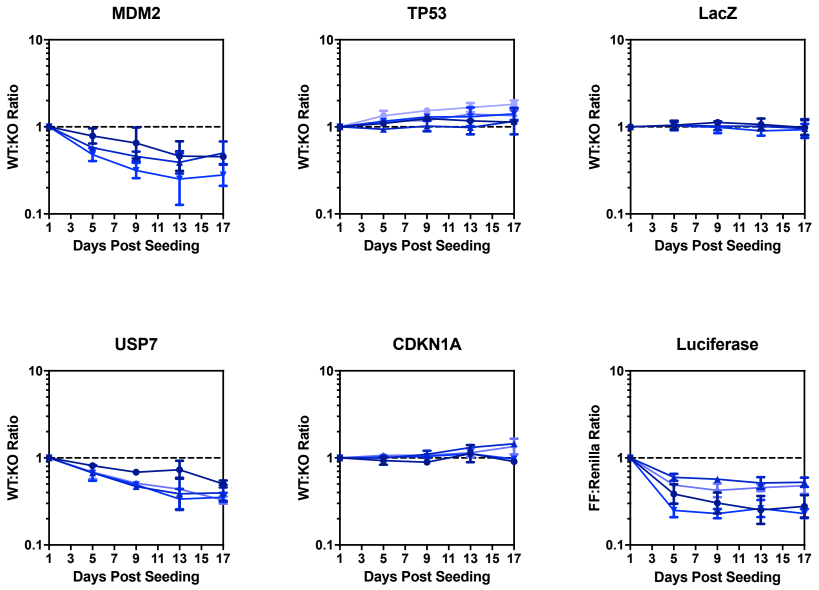

RKO

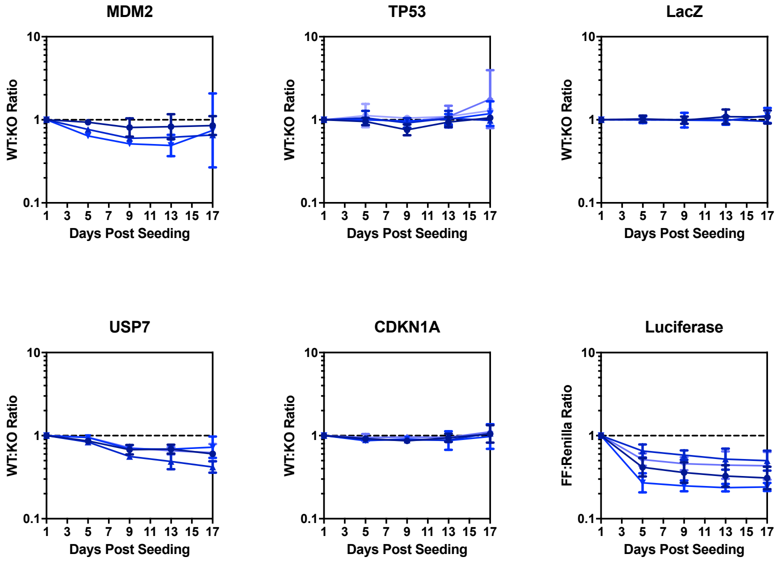

Figure S13

Figure 1f

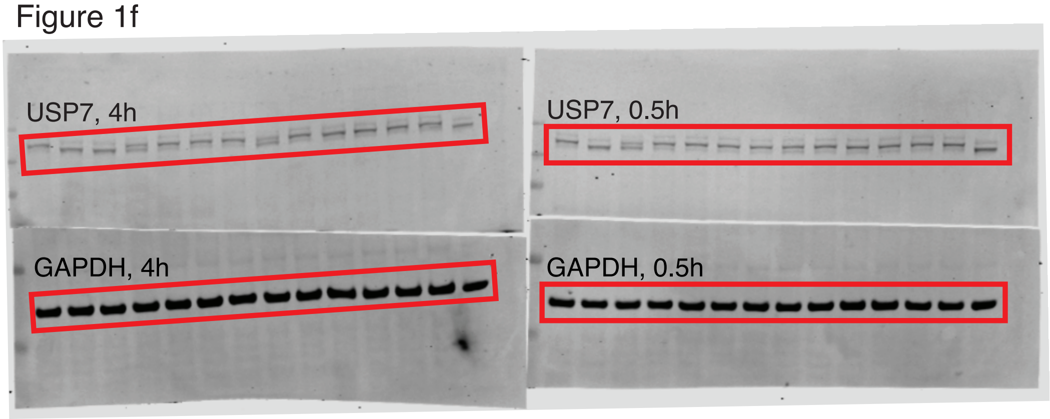

Figures 2a and 2c (partial)

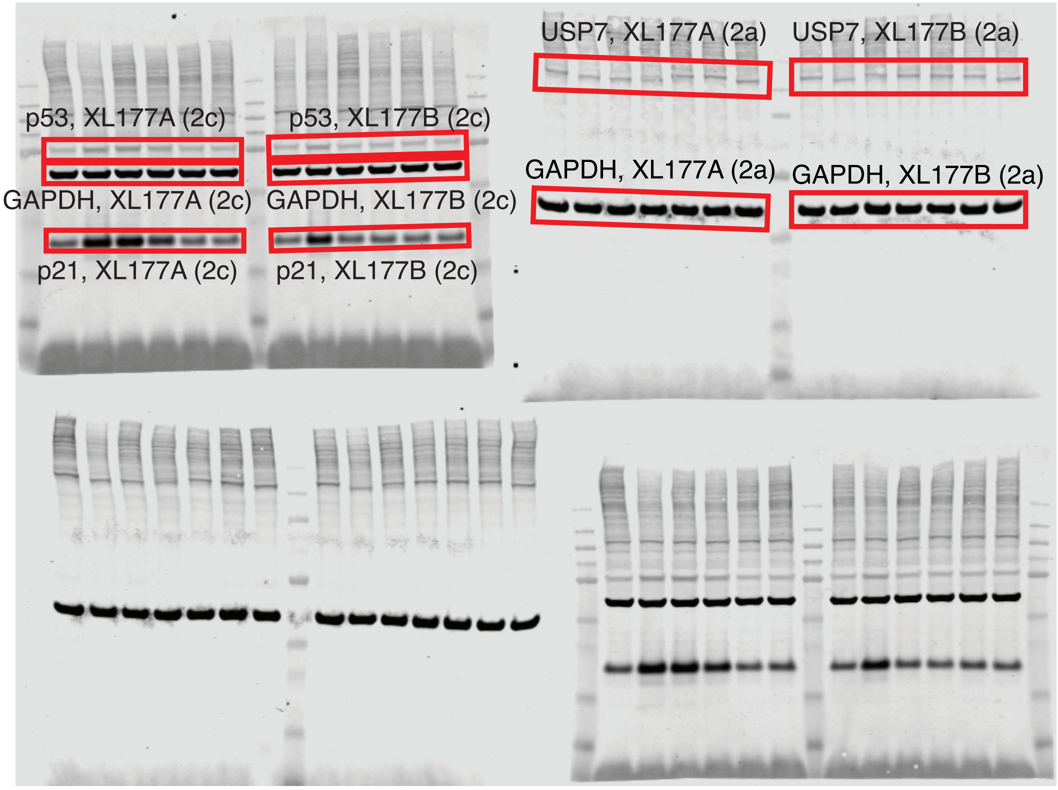

Figure S5

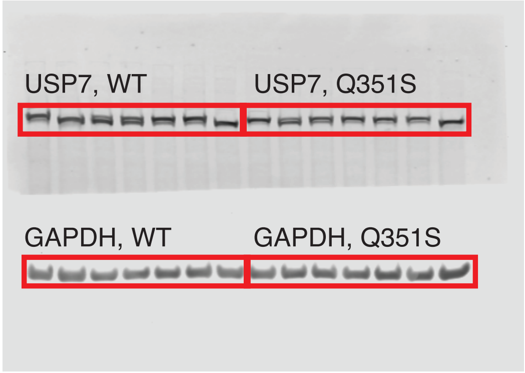

Figure 2c (partial)

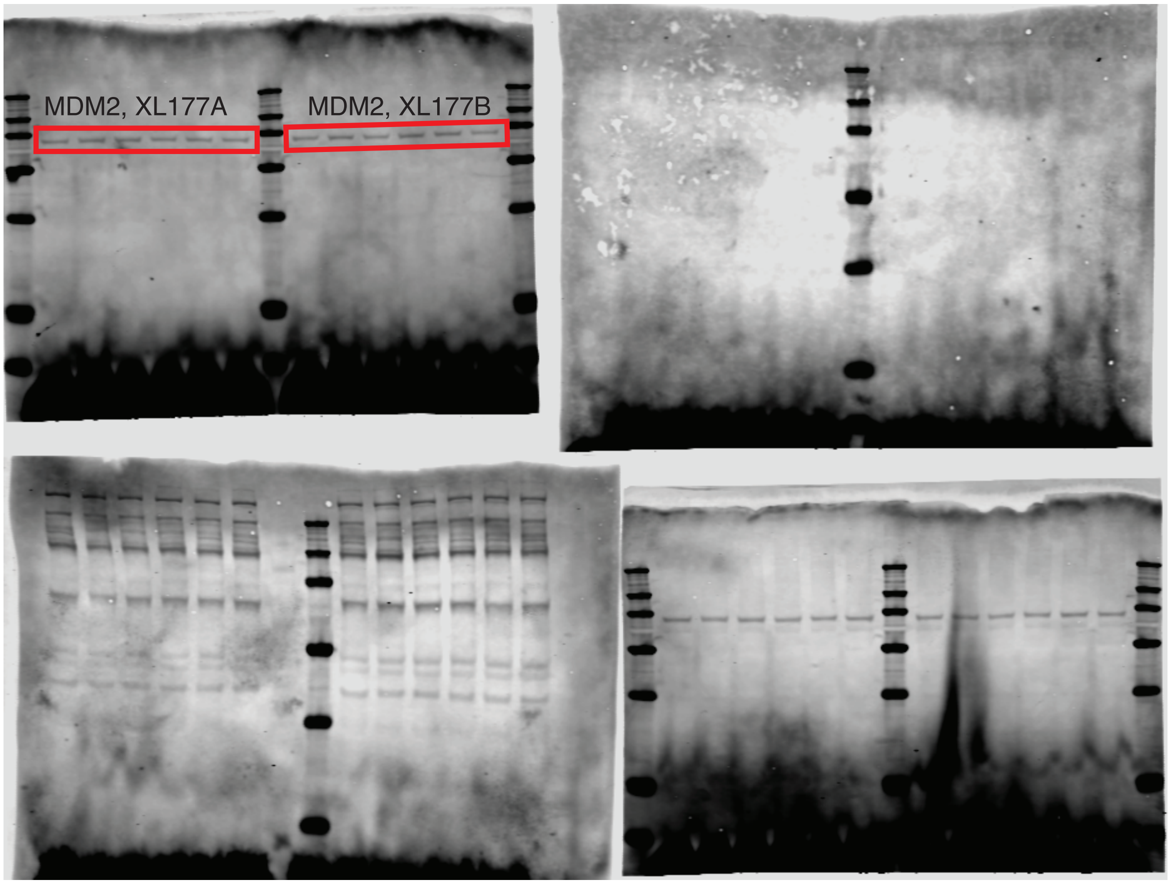

Figure 2b

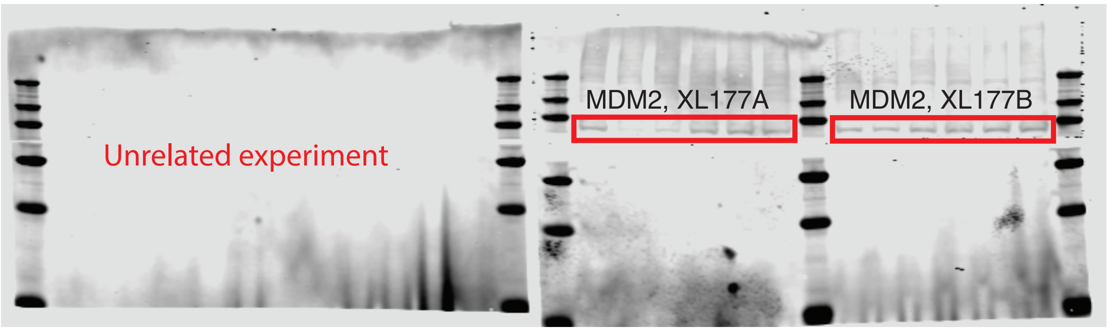

Figure 2b

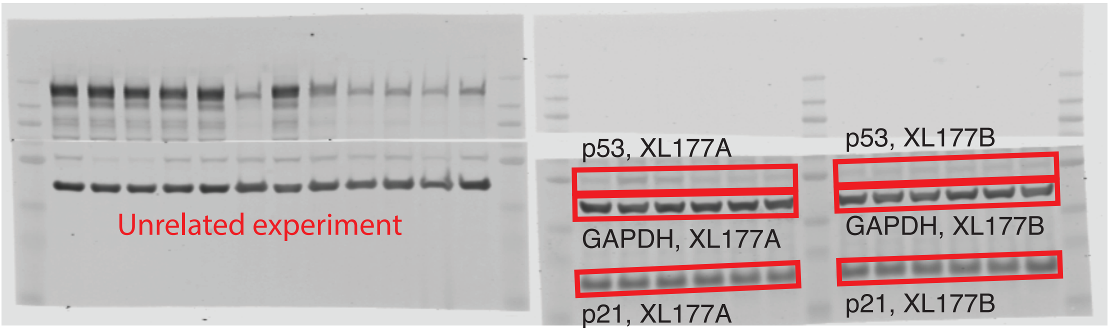

Figure S14

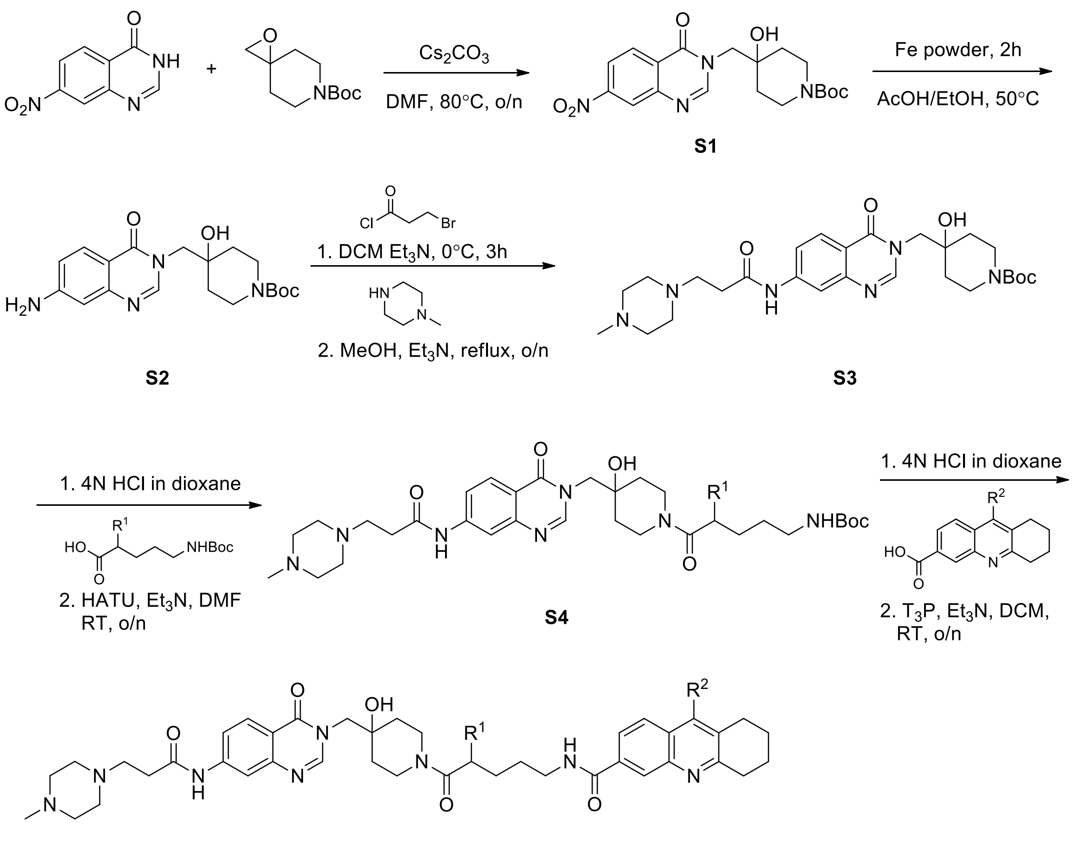

	XL177A	XL177B	XL112	XL041	XL058
R^1	(*S*)-Bn	(*R*)-Bn	(*rac*)-Bn	H	(*rac*)-Bn
R^2	Cl	Cl	Cl	Cl	H

Figure S15

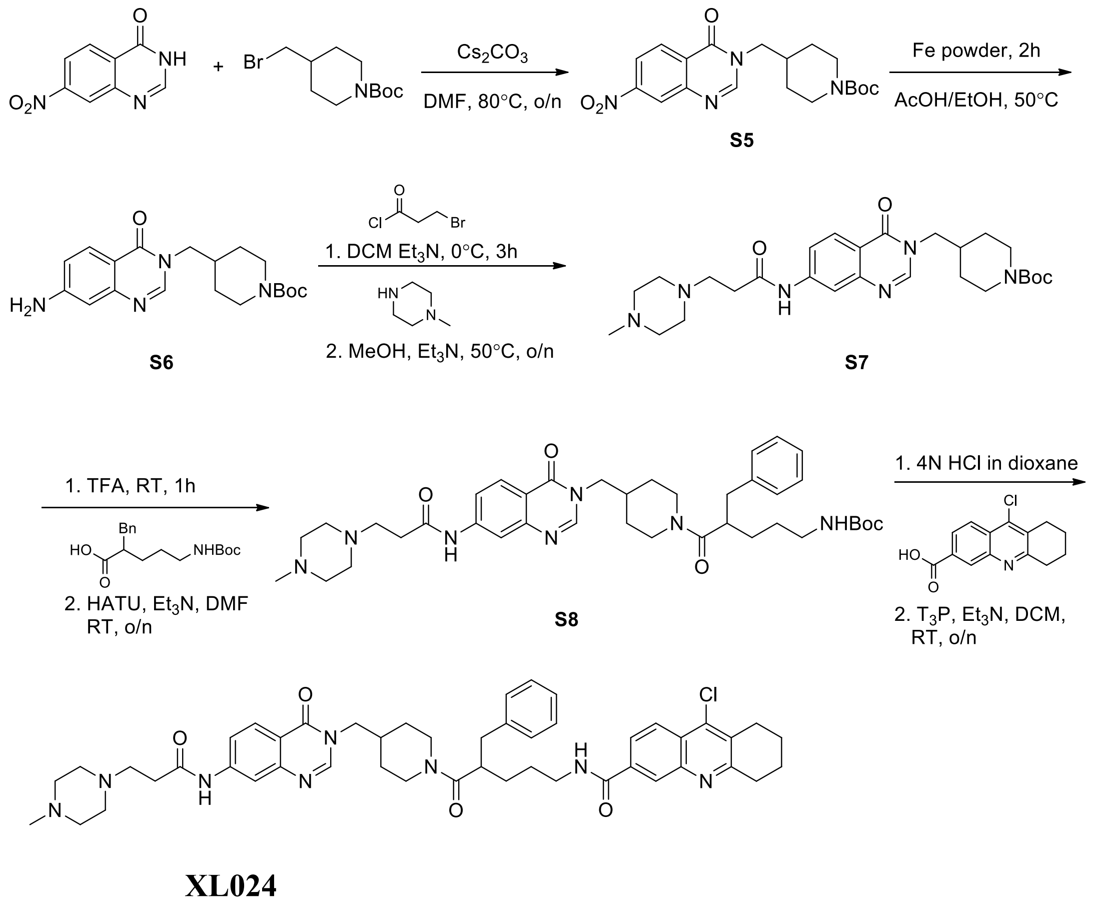

Cs_2CO_3
DMF, 80°C, o/n
S5
Fe powder, 2h
AcOH/EtOH, 50°C
S6
1. DCM Et_3N, 0°C, 3h
2. MeOH, Et_3N, 50°C, o/n
S7
1. TFA, RT, 1h
2. HATU, Et_3N, DMF RT, o/n
S8
1. 4N HCl in dioxane
2. T_3P, Et_3N, DCM, RT, o/n
XL024

Figure S16

Supplementary Synthetic Methods

Synthesis of XL177A, XL177B, XL112, XL041, and XL058 (see Figure S15):

Step 1 (Synthesis of **S1**): 7-nitroquinazolin-4(*3H*)-one (1.55g, 8.1mmol) and *tert*-butyl-1-oxa-6-azaspiro[2.5]octane-6-carboxylate (1.90g, 8.9mmol) were added into 20mL DMF. Cesium carbonate (7.82g, 24.0mmol) was added in one portion. The mixture was heated at 80°C overnight. The mixture was diluted with EtOAc, then washed with sat. NaCl. Combined organic layer was concentrated under reduced pressure. The crude product was purified by flash chromatography (EtOAc: hexanes: 50%-70%) to afford 2.42g **S1** (75%). ^{1}H NMR (500 MHz, $CDCl_3$) δ 8.54 (d, *J* = 2.1 Hz, 1H), 8.44 (t, *J* = 8.6 Hz, 1H), 8.26 (dd, *J* = 8.8, 2.2 Hz, 1H), 8.20 (s, 1H), 4.10 (s, 2H), 3.88 (s, 2H), 3.14 (t, *J* = 11.6 Hz, 2H), 1.73 – 1.48 (m, 5H), 1.44 (s, 9H). LCMS (ESI) *m*/*z* 304.97 (M＋H－Boc) [$(M+H)^+$ $C_{19}H_{25}N_4O_6^+$ calcd for 405.18]

Step 2 (Synthesis of **S2**): Compound **S1**(2.4g, 6.0mmol) was suspended in 20mL solvent (EtOH/AcOH=1:1). 4 eq. of Fe powder was added in portions. The mixture was stirred for 1 hour at 55°C. Then the reaction was cooled down to room temperature, and filtered through a pad of Celite. The filtrate was concentrated under reduced pressure to afford the crude product, which was then purified by flash chromatography (10%MeOH in EtOAc) to afford 2.1g product **S2** (93%) ^{1}H NMR (500 MHz, DMSO) δ 8.04 (s, 1H), 7.79 (d, *J* = 8.7 Hz, 1H), 6.72 (dd, *J* = 8.7, 1.9 Hz, 1H), 6.61 (d, *J* = 2.0 Hz, 1H), 6.09 (s, 2H), 4.87 (s, 1H), 3.89 (s, 2H), 3.64 (d, *J* = 12.0 Hz, 2H), 3.05 (s, 2H), 1.54 – 1.24 (m, 13H).LCMS (ESI) *m*/*z* 374.97 [$(M+H)^+$ $C_{19}H_{27}N_4O_4^+$ calcd for 375.20]

Step 3 (Synthesis of **S3**): Compound **S2** (2.1g, 5.6mmol) was dissolved in anhydrous 10mL dichloromethane under N_2 at 0°C. 3.0 eq. of Et_3N was added. Then 3-bromopropionyl chloride (1.15g, 6.7mmol) was added dropwise. The mixture was stirred at 0°C for 1 hour, then quenched with MeOH, and concentrated under reduced pressure. The solid residue was directly used for the following step without further purification. The crude product from last step was dissolved in 10mL MeOH, then 3.0eq of Et_3N was added. Into the stirred mixture was added 1-methylpiperazine (0.67g, 6.7mmol) dropwise. After the addition completed, the mixture was stirred for 1 hour at 50°C. Then the reaction mixture was cooled down to room temperature and concentrated under reduced pressure, then directly subjected to HPLC purification (MeOH/H_2O with 4‰ TFA) to afford 2.1g product **S3** (73% in two steps) ^{1}H NMR (500 MHz, MeOD) δ 8.28 (s, 1H), 8.20 (d, *J* = 8.8 Hz, 1H), 8.12 (d, *J* = 1.9 Hz, 1H), 7.69 (dd, *J* = 8.7, 2.0 Hz, 1H), 4.11 (s, 2H), 3.82 (d, *J* = 13.4 Hz, 2H), 3.23 (m, 2H), 2.85 (t, *J* = 7.0 Hz, 2H), 2.79 – 2.50 (m, 10H), 2.37 (s, 3H), 1.72 – 1.62 (m, 2H), 1.50 (d, *J* = 17.4 Hz, 11H). LCMS (ESI) *m*/*z* 529.08 [$(M+H)^+$ $C_{27}H_{41}N_6O_5^+$ calcd for 529.31].

Step 4 (Synthesis of **S4** (R^1=(*S*)-Bn, R^2=Cl)): **S3** (0.53g, 1.0mmol) was dissolved in 3mL DCM, then 5mL 4M HCl in 1,4-dioxane was added in portions. The solution was stirred for 1 hour at room temperature. Then the

mixture was concentrated under reduced pressure, and left on high vacuum overnight to remove residual acid. Then the product (0.11g, 0.25mmol) was dissolved in 3mL DMF, and basified by adding 10 eq of Et_3N. Sequentially, (*S*)-2-benzyl-5-((tert-butoxycarbonyl)amino)pentanoic acid (0.11g, 0.35mmol) and HATU (0.16g, 0.4mmol) were added to the solution. The resultant solution was stirred overnight. Then the mixture was directly subjected to HPLC purification (MeOH/H_2O with 4‰ TFA) to afford 183mg **S4** (quantitative) ^{1}H NMR (500 MHz, DMSO) δ 10.51 (s, 1H), 8.16-8.04 (m, 2H, conformer), 8.01 (dd, *J* = 5.0, 1.9 Hz, 1H), 7.61 (ddd, *J* = 9.8, 8.3, 2.0 Hz, 1H), 7.29 – 7.19 (m, 2H), 7.19 – 7.07 (m, 3H), 6.76 (m, 1H), 4.84 (s, 1H), 4.11 (m, 1H), 4.05 – 3.87 (m, 2H), 3.80 (d, *J* = 13.7 Hz, 1H), 3.67 – 3.49 (m, 3H), 3.16 – 3.03 (m, 2H), 2.98 (s, 1H), 2.85 (m, 3H), 2.77 – 2.58 (m, 5H), 2.55 (m, 3H), 2.22 (s, 3H), 1.61 – 1.44 (m, 1H), 1.43 – 1.33 (m, 9H, conformer), 1.33 – 1.22 (m, 3H), 1.22 – 1.02 (m, 4H), 0.39 (m, 1H). LCMS (ESI) *m/z* 718.00 [$(M+H)^+$ 718.43 calcd for $C_{39}H_{56}N_7O_6^+$]

Step 5 (Synthesis of **XL177A**): **S4** (0.18g, 0.25mmol) was dissolved in 4M HCl in 1,4-dioxane, and stirred for 1h at room temperature. Then the mixture was concentrated under reduced pressure, and left on high vacuum overnight to remove residual solvent. Then the product (0.16g, 0.25mmol) was dissolved in 5mL DCM with 10 eq. of Et_3N. Into the solution were added 9-chloro-5,6,7,8-tetrahydroacridine-3-carboxylic acid (0.10g, 0.4mmol) and T3P (50% in EtOAc) (0.42g, 1.3mmol). The solution was stirred at room temperature under nitrogen overnight. Then the mixture was concentrated under reduced pressure, and purified sequentially by flash chromatography (MeOH in EtOAc: 0%-70%) and HPLC (MeOH/H_2O with 4‰ TFA) to afford 98mg XL177A (46%).

XL177A

(S)-N-(4-benzyl-5-(4-hydroxy-4-((7-(3-(4-methylpiperazin-1-yl)propanamido)-4-oxoquinazolin-3(4H)-yl)methyl)piperidin-1-yl)-5-oxopentyl)-9-chloro-5,6,7,8-tetrahydroacridine-3-carboxamide. ^{1}H NMR (500 MHz, DMSO) δ 10.50 (d, *J* = 5.5 Hz, 1H), 8.85 – 8.69 (m, 1H), 8.46 (d, *J* = 14.0 Hz, 1H), 8.22 – 7.93 (m, 5H), 7.61 (dd, *J* = 16.4, 9.3 Hz, 1H), 7.17 (ddt, *J* = 32.3, 19.2, 7.4 Hz, 5H), 4.82 (d, *J* = 4.5 Hz, 1H), 4.20 – 3.93 (m, 1H), 3.93 – 3.74 (m, 2H), 3.62 (m, 2H), 3.30 (m, 4H, overlapped with H_2O), 3.20 – 3.09 (m, 2H), 3.05 (m, 2H), 2.96 (m, 2H), 2.85 (m, 1H), 2.80 – 2.70 (m, 2H), 2.66 (m, 4H), 2.58 – 2.51 (m, 3H), 2.17 (s, 4H), 1.88 (d, *J* = 3.2 Hz, 4H), 1.71 – 1.35 (m, 5H), 1.34 – 1.02 (m, 4H), 0.41 (t, *J* = 10.5 Hz, 1H). ^{13}C NMR (126 MHz, DMSO) δ 172.24/172.19 (conformer), 170.98, 165.30/165.25 (conformer), 160.38/160.33 (conformer), 160.08/160.01(conformer), 149.32/149.27(conformer), 148.95, 145.59/145.56(conformer), 144.24, 139.97, 139.81/139.75(conformer), 135.19/135.17(conformer), 130.07/130.02(conformer), 128.93/128.79(conformer), 128.12/128.00(conformer), 127.43/127.37(conformer), 127.26/127.20(conformer), 125.99/125.94(conformer), 125.78, 125.37/125.32(conformer), 123.39, 118.25/118.20(conformer) 116.56/116.45(conformer), 114.67,

69.16/69.08(conformer), 54.62, 53.53/53.33(conformer), 53.44, 52.20, 45.54, 41.47/41.24(conformer), 40.90/40.68(conformer), 38.65, 37.01/36.96(conformer), 35.22, 34.63, 34.47, 34.26, 33.96, 33.56, 30.46, 29.66, 28.98, 27.05, 26.76, 21.86. LCMS (ESI) *m/z* 860.82 [$(M+H)^+$; $C_{48}H_{58}ClN_8O_5^+$ calcd for 861.42]

XL177B was synthesized according to procedure A using (*R*)-2-benzyl-5-((tert-butoxycarbonyl)amino)pentanoic acid in step 4 and 9-chloro-5,6,7,8-tetrahydroacridine-3-carboxylic acid in step 5.

(R)-N-(4-benzyl-5-(4-hydroxy-4-((7-(3-(4-methylpiperazin-1-yl)propanamido)-4-oxoquinazolin-3(4H)-yl)methyl)piperidin-1-yl)-5-oxopentyl)-9-chloro-5,6,7,8-tetrahydroacridine-3-carboxamide. ^1H NMR (500 MHz, DMSO) δ 10.49 (d, *J* = 5.6 Hz, 1H), 8.82 – 8.69 (m, 1H), 8.46 (d, *J* = 14.0 Hz, 1H), 8.20 – 7.95 (m, 5H), 7.66 – 7.55 (m, 1H), 7.30 – 7.05 (m, 5H), 4.82 (d, *J* = 4.8 Hz, 1H), 4.06 (dd, *J* = 62.9, 12.9 Hz, 1H), 3.95 – 3.75 (m, 1H), 3.61 (m, 2H,), 3.31 – 3.21 (m, 4H, overlapped with H_2O), 3.12 (m, 2H), 3.04 (m, 2H), 2.95 (m, 2H), 2.83 (m, 1H), 2.79 – 2.70 (m, 2H), 2.70 – 2.60 (m, 4H), 2.53 (m, 3H), 2.20 (s, 4H), 1.94 – 1.81 (m, 4H), 1.70 – 1.34 (m, 5H), 1.34 – 1.00 (m, 4H), 0.40 (dt, *J* = 12.0, 9.1 Hz, 1H).^{13}C NMR (126 MHz, DMSO) δ 172.75/172.70（conformer), 171.45, 165.81/165.77 (conformer), 160.89/160.84 (conformer), 160.58/160.52 (conformer), 149.82/149.78 (conformer), 149.45, 146.09/146.06 (conformer), 144.74, 140.47, 140.32/140.25 (conformer), 135.69/135.67 (conformer), 130.58/130.53 (conformer), 129.43/129.29 (conformer), 128.63/128.51 (conformer), 127.93/127.87 (conformer), 127.77/127.70 (conformer), 126.50/126.44 (conformer), 126.28, 125.87/125.82 (conformer), 123.90, 118.76, 117.07/116.96 (conformer), 115.18, 69.67/69.59 (conformer), 54.98, 54.04, 53.88, 52.52, 45.83, 41.97/41.75 (conformer), 41.41/41.19 (conformer), 39.15, 37.52/37.46 (conformer), 35.73, 35.13, 34.97, 34.74, 34.46, 34.06, 30.97, 30.16, 29.48, 27.56, 27.26, 22.36. LCMS (ESI) *m/z* 860.72 [$(M+H)^+$ $C_{48}H_{58}ClN_8O_5^+$ calcd for 861.42]

XL112 was synthesized according to procedure A using (*rac*)-2-benzyl-5-((tert-butoxycarbonyl)amino)pentanoic acid in step 4 and 9-chloro-5,6,7,8-tetrahydroacridine-3-carboxylic acid in step 5.

N-(4-benzyl-5-(4-hydroxy-4-((7-(3-(4-methylpiperazin-1-yl)propanamido)-4-oxoquinazolin-3(4H)-yl)methyl)piperidin-1-yl)-5-oxopentyl)-9-chloro-5,6,7,8-tetrahydroacridine-3-carboxamide ^1H NMR (500 MHz, DMSO) δ 10.50 (d, *J* = 5.5 Hz, 1H), 8.82 – 8.70 (m, 1H), 8.46 (d, *J* = 13.8 Hz, 1H), 8.22 – 7.93 (m, 5H), 7.60 (dd, *J* = 16.4, 9.4 Hz, 1H), 7.29 – 7.03 (m, 5H), 4.82 (s, 1H), 4.06 (dd, *J* = 62.6, 12.8 Hz, 1H), 3.94 – 3.73 (m, 1H), 3.62 (m, 2H), 3.28 (m, 4H, overlapped with H_2O), 3.13 (t, *J* = 11.0 Hz, 2H), 3.05 (m, 2H), 2.96 (d, *J* = 11.0 Hz, 2H), 2.85 (m, 1H), 2.79 – 2.71 (m, 2H), 2.70 – 2.60 (m, 4H), 2.53 (m, 3H), 2.14 (s, 4H), 1.88 (d, *J* = 3.1 Hz, 4H), 1.71 – 1.34 (m, 5H), 1.33 – 1.03 (m, 4H), 0.39 (m, 1H). ^{13}C NMR (126 MHz, DMSO) δ 172.74/172.69(conformer), 171.51, 165.80/165.76(conformer), 160.89/160.84(conformer), 160.58/160.52(conformer), 149.82/149.78(conformer), 149.46, 146.10/146.07(conformer), 144.75, 140.47,

140.32/140.25(conformer), 135.68, 130.58/130.53(conformer), 129.43/129.29(conformer), 128.63/128.51(conformer), 127.93/127.87(conformer), 127.77/127.70(conformer), 126.50/126.44(conformer), 126.29, 125.87/125.83(conformer), 123.90, 118.75, 117.07/116.95(conformer), 115.17, 69.67/69.59(conformer), 55.22, 54.04/53.84(conformer), 53.99, 52.82, 46.19, 41.97/41.74,(conformer) 41.41/41.19(conformer), 39.15, 37.51/37.46(conformer), 35.73, 35.13, 34.97, 34.78, 34.47, 34.06, 30.97, 30.16, 29.48, 27.56, 27.26/27.23(conformer), 22.36. LCMS (ESI) *m/z* 860.72 [$(M+H)^+$; $C_{48}H_{58}ClN_8O_5^+$ calcd for 861.42]

XL058 was synthesized according to procedure A using (*rac*)-2-benzyl-5-((tert-butoxycarbonyl)amino)pentanoic acid in step 4 and 5,6,7,8-tetrahydroacridine-3-carboxylic acid in step 5.

N-(4-benzyl-5-(4-hydroxy-4-((7-(3-(4-methylpiperazin-1-yl)propanamido)-4-oxoquinazolin-3(4H)-yl)methyl)piperidin-1-yl)-5-oxopentyl)-5,6,7,8-tetrahydroacridine-3-carboxamide ^{1}H NMR (500 MHz, DMSO) δ 10.50 (d, *J* = 4.4 Hz, 1H, conformer), 8.74 – 8.60 (m, 1H), 8.40 (d, *J* = 14.3 Hz, 1H, conformer), 8.11 (m, 2H), 8.07 – 7.96 (m, 2H), 7.92 – 7.81 (m, 2H), 7.69 – 7.52 (m, 1H), 7.16 (m, 5H), 4.82 (s, 1H, conformer), 4.06 (dd, *J* = 61.0, 12.9 Hz, 1H, conformer), 3.88 (q, *J* = 13.9 Hz, 1H), 3.71 (dd, *J* = 88.4, 13.6 Hz, 3H), 3.27 (m, 2H), 3.13 (m, 2H), 3.03 (dd, *J* = 7.9, 4.8 Hz, 2H), 2.96 (m, 2H), 2.83 (m, 1H), 2.80 – 2.69 (m, 2H), 2.69 – 2.59 (m, 4H), 2.59 – 2.52 (m, 3H), 2.16 (d, *J* = 18.6 Hz, 3H), 1.98 – 1.87 (m, 2H), 1.87 – 1.77 (m, 2H), 1.60 (d, *J* = 29.3 Hz, 1H), 1.43 (m, 4H), 1.35 – 1.00 (m, 4H), 0.40 (t, *J* = 10.7 Hz, 1H). ^{13}C NMR (126 MHz, DMSO) δ 172.76/172.70(conformer), 171.52, 166.30, 160.61/160.52(conformer), 160.32/160.30(conformer), 149.84/149.79(conformer), 149.46, 145.88/145.85(conformer), 144.75, 140.49, 140.25, 134.81, 134.75, 132.73/132.70(conformer), 129.44/129.30(conformer), 128.62/128.51(conformer), 127.78/127.75(conformer), 127.62, 127.42/127.37(conformer), 126.49/126.44(conformer), 124.42/124.38(conformer), 118.76, 117.07/116.97(conformer), 115.17, 69.67/69.59(conformer), 55.23, 54.04/53.84(conformer), 54.00, 52.84, 46.20, 41.97/41.74(conformer), 41.40/41.17(conformer), 39.18, 37.46, 35.72, 35.13, 34.95, 34.79, 34.47, 33.46, 30.97, 30.17, 29.03, 27.31, 23.05, 22.75.LCMS (ESI) *m/z* 827.61 [$(M+H)^+$ $C_{48}H_{59}N_8O_5^+$ calcd for 827.46]

XL041 was synthesized according to procedure A using 5-((tert-butoxycarbonyl)amino)pentanoic acid in step 4 and 9-chloro-5,6,7,8-tetrahydroacridine-3-carboxylic acid in step 5.

9-chloro-N-(5-(4-hydroxy-4-((7-(3-(4-methylpiperazin-1-yl)propanamido)-4-oxoquinazolin-3(4H)-yl)methyl)piperidin-1-yl)-5-oxopentyl)-5,6,7,8-tetrahydroacridine-3-carboxamide ^{1}H NMR (500 MHz, DMSO) δ 10.60 (s, 1H), 8.79 (t, *J* = 5.0 Hz, 1H), 8.46 (s, 1H), 8.22 (s, 1H), 8.15 (d, *J* = 8.7 Hz, 1H), 8.06 (dd, *J* = 14.6, 8.7 Hz, 3H), 7.64 (d, *J* = 8.8 Hz, 1H), 4.11 – 3.90 (m, 4H), 3.65 (d, *J* = 13.0 Hz, 1H), 3.38 – 3.20 (m, 6H), 3.07 (m,5H), 2.96 (m, 4H), 2.77 (m,6H), 2.36 (m, 2H), 1.88 (s, 3H), 1.55 (d, *J* = 26.5 Hz, 6H), 1.48 – 1.31 (m, 4H). ^{13}C NMR (126 MHz, DMSO) δ 170.76, 170.28, 165.81, 160.83, 160.63, 149.94, 149.38, 145.93, 144.60, 140.44,

135.78, 130.56, 127.77, 127.74, 126.26, 125.85, 123.90, 118.84, 117.12, 115.34, 69.80, 53.86, 52.38, 51.73, 49.40, 42.61, 41.48, 39.60, 37.41, 35.58, 34.82, 33.98, 33.18, 32.45, 29.17, 27.54, 22.89, 22.33. LCMS (ESI) *m/z* 771.47 [(M+H)$^+$; $C_{41}H_{52}ClN_8O_5^+$calcd for 771.37]

Synthesis of XL024 (see Figure S16):

Step 1 (Synthesis of **S5**): 7-nitroquinazolin-4(*3H*)-one (0.77g, 4.0mmol) and tert-butyl 4-(bromomethyl)piperidine-1-carboxylate (1.11g, 4.0mmol) were added into 10mL DMF. Cesium carbonate (3.90g, 12.0mmol) was added in one portion. The mixture was heated at 80°C overnight. The mixture was diluted with EtOAc, then washed with sat. NaCl. Combined organic layer was concentrated under reduced pressure. The crude product was purified by flash chromatography (EtOAc in hexanes: 40%-100%) to afford 1.14g **S5** (73%). ^{1}H NMR (500 MHz, $CDCl_3$) δ 8.54 (d, *J* = 2.0 Hz, 1H), 8.45 (d, *J* = 8.8 Hz, 1H), 8.26 (dd, *J* = 8.8, 2.1 Hz, 1H), 8.04 (s, 1H), 4.14 (br, 2H), 3.89 (br, 2H), 2.65 (m, 2H), 2.05 (m,1H), 1.65 (d, *J* = 12.4 Hz, 2H), 1.43 (s, 9H), 1.24 (qd, *J* = 12.6, 4.3 Hz, 2H).LCMS (ESI) *m/z* 332.87 (M＋H－*t*-Butyl) [(M+H)$^+$ $C_{19}H_{25}N_4O_5^+$ calcd for 389.18]

Step 2 (Synthesis of **S6**): Compound **S5** (1.14g, 2.9mmol) was suspended in 10mL solvent (EtOH/AcOH=1:1). 4 eq. of Fe powder was added in portions. The mixture was stirred for 2 hour at 50°C. Then the reaction was cooled down to room temperature, and filtered through a pad of Celite. The filtrate was concentrated under reduced pressure to afford the crude product, which was then purified by flash chromatography (MeOH in EtOAc: 0% to 70%) to afford 1.07g product **S6** (quant.) ^{1}H NMR (500 MHz, $CDCl_3$) δ 8.09 – 8.03 (dd, *J* = 7.5, 1.5 Hz, 1H), 7.85 (s, 1H), 6.80 – 6.74 (m, 2H), 4.23 (br, 2H), 4.10 (br, 2H), 3.78 (br, 2H), 2.63 (m, 2H), 2.06 – 1.97 (m, 1H), 1.64 (m, 2H), 1.45 – 1.39 (s, 9H), 1.20 (qd, *J* = 12.5, 4.0 Hz, 2H). LCMS (ESI) *m/z* 302.97 (M＋H－*t*-Butyl) [(M+H)$^+$ $C_{19}H_{27}N_4O_3^+$ calcd for 359.21]

Step 3 (Synthesis of **S7**): Compound **S6** (0.6g, 1.68mmol) was dissolved in 5mL anhydrous dichloromethane under N_2 at 0°C. 3.0 eq. of Et_3N was added. Then 3-bromopropionyl chloride (0.35g, 2.1mmol) was added dropwise. The mixture was stirred at 0°C for 3 hours, then quenched with MeOH, and concentrated under reduced pressure. The solid residue was directly used for the following step without further purification. The crude product from last step was dissolved in 10mL MeOH, then 3.0eq of Et_3N was added. Into the stirred mixture was added 1-methylpiperazine (0.26g, 2.52mmol) dropwise. After the addition completed, the mixture was stirred at 50°C overnight. Then the reaction mixture was cooled down to room temperature, then directly subjected to flash chromatography purification (MeOH in EtOAc: 0% to 70%) to afford 0.72g product **S7** (83% in two steps) ^{1}H NMR (500 MHz, $CDCl_3$) δ 11.42 (s, 1H), 8.22 (d, *J* = 8.7 Hz, 1H), 7.91 (s, 1H), 7.84 (d, *J* = 2.0 Hz, 1H), 7.72 (dd, *J* = 8.7, 2.0 Hz, 1H), 4.11 (s, 2H), 3.83 (s, 2H), 2.74 (m, 4H), 2.64 (m, 4H), 2.59 – 2.46 (m, 4H), 2.36 (s, 3H),

2.13 – 1.98 (m, 1H), 1.65 (m, 4H), 1.43 (s, 9H), 1.21 (qd, *J* = 12.4, 4.2 Hz, 2H). LCMS (ESI) *m/z* 512.98 [$(M+H)^+$ $C_{27}H_{41}N_6O_4^+$ calcd for 513.32].

Step 4 (Synthesis of **S8**): **S7** (0.17g, 0.32mmol) was dissolved in 3mL TFA. The solution was stirred for 1 hour at room temperature. Then the mixture was concentrated under reduced pressure, and left on high vacuum overnight to remove residual acid. Then the product (0.14g, 0.32mmol) was dissolved in 3mL DMF, and basified by adding 10 eq of Et_3N. Into the solution 2-benzyl-5-((tert-butoxycarbonyl)amino)pentanoic acid (0.14g, 0.48mmol) and HATU (0.24g, 0.64mmol) sequentially The resultant solution was stirred overnight. Then the mixture was directly subjected to flash chromatography purification (MeOH in EtOAc: 0% to 70%) to afford 0.23g **S8** (quantitative) ^{1}H NMR (500 MHz, DMSO) δ 10.52 (s, 1H), 8.27 and 8.13 (s, 1H, conformer), 8.07 (d, *J* = 8.5 Hz, 1H, conformer), 8.04 – 7.99 (d, *J* = 7.5 Hz, 1H, conformer), 7.67 – 7.58 (m, 1H), 7.25 (m, 2H), 7.19 – 7.08 (m, 3H), 6.83 – 6.71 (m, 1H), 4.37 (d, *J* = 12.5 Hz, 1H, conformer), 3.82 (m, 2H), 3.64 – 3.57 (dd, *J* = 13.5, 7.5 Hz, 1H, conformer), 3.05 (m, 1H), 2.96 – 2.80 (m, 2H), 2.80 – 2.68 (m, 2H), 2.62 (m, 3H), 2.54 (m, 2H), 2.37 (m, 8H), 2.16 (s, 3H), 2.00 – 1.81 (m, 1H), 1.52 (m, 1H), 1.37 (m, 11H), 1.31 – 1.19 (m, 2H), 1.13 – 0.98 (m, 1H), 0.71 and -0.03 (dd, *J* = 21.2, 11.8 Hz, 1H, conformer). LCMS (ESI) *m/z* 701.90 [$(M+H)^+$ $C_{39}H_{56}N_7O_5^+$ calcd for 702.43]

Step 5 (Synthesis of **XL024**): **S8** (0.13g, 0.18mmol) was dissolved in 4M HCl in 1,4-dioxane, and stirred for 1h at room temperature. Then the mixture was concentrated under reduced pressure, and left on high vacuum overnight to remove residual solvent. Then the product (0.11g, 0.18mmol) was dissolved in 5mL anhydrous DCM with 10 eq. of Et_3N. Into the solution was added 9-chloro-5,6,7,8-tetrahydroacridine-3-carboxylic acid (0.05g, 0.2mmol), and T3P (50% in EtOAc) (0.17g, 0.54mmol). The solution was stirred at room temperature under nitrogen overnight. Then the mixture was concentrated under reduced pressure, and purified sequentially by flash chromatography (MeOH in EtOAc: 0% to 70%) and HPLC (MeOH/H_2O with 4‰ TFA) to afford 50mg XL024 (33%).

(4-benzyl-5-(4-((7-(3-(4-methylpiperazin-1-yl)propanamido)-4-oxoquinazolin-3(4H)-yl)methyl)piperidin-1-yl)-5-oxopentyl)-9-chloro-5,6,7,8-tetrahydroacridine-3-carboxamide ^{1}H NMR (500 MHz, DMSO) δ 10.52 (d, *J* = 4.2 Hz, 1H, conformer), 8.78 (dt, *J* = 18.3, 5.4 Hz, 1H, conformer), 8.48 (d, *J* = 18.1 Hz, 1H, conformer), 8.23 – 8.11 (m, 2H), 8.11 – 7.99 (m, 3H), 7.62 (m, 1H), 7.19 (m, 5H), 4.39 (dd, *J* = 39.4, 12.8 Hz, 1H, conformer), 3.78 (m, 2H), 3.61 (ddd, *J* = 43.3, 13.4, 7.0 Hz, 1H), 3.29 (m, 2H), 3.13 (s, 1H), 3.06 (m, 2H), 2.99 (m, 2H), 2.88 – 2.71 (m, 2H), 2.71 – 2.59 (m, 4H), 2.58 – 2.52 (m, 3H), 2.39 (m, 4H), 2.15 (s, 3H), 2.00 – 1.83 (m, 5H), 1.74 – 1.35 (m, 6H), 1.23 (m, 1H), 1.05 (td, *J* = 23.0, 11.6 Hz, 1H), 0.72 (dt, *J* = 12.3, 8.8 Hz, 1H). ^{13}C NMR (126 MHz, DMSO) δ 172.78/172.70(conformer), 171.53, 165.81/165.75(conformer), 160.91, 160.17, 149.45, 148.92,

146.06, 144.77, 140.50, 140.33/140.28(conformer), 135.68, 130.59, 129.58/129.32(conformer), 128.69/128.51(conformer), 127.89/127.87(conformer), 127.54/127.49(conformer), 126.51/126.44(conformer), 126.32/126.29(conformer), 125.87/125.84(conformer), 123.96/123.91(conformer), 118.94, 117.03/116.98(conformer), 115.25, 55.23, 54.00, 52.84, 50.97/50.80(conformer), 46.20, 45.31, 44.72, 42.09/41.83(conformer), 41.54, 41.16, 39.01, 35.56/35.39(conformer), 34.79, 34.07, 30.92, 30.30, 29.98, 29.61, 29.35, 27.56, 27.28/27.18(conformer), 22.36. LCMS (ESI) *m*/*z* 844.82 [$(M+H)^+$ $C_{48}H_{58}ClN_8O_4^+$ calcd for 845.43]
